# Oxidative Stress and Neuroinflammation as a Pivot in Drug Abuse. A Focus on the Therapeutic Potential of Antioxidant and Anti-Inflammatory Agents and Biomolecules

**DOI:** 10.3390/antiox9090830

**Published:** 2020-09-04

**Authors:** Pablo Berríos-Cárcamo, Mauricio Quezada, María Elena Quintanilla, Paola Morales, Marcelo Ezquer, Mario Herrera-Marschitz, Yedy Israel, Fernando Ezquer

**Affiliations:** 1 Center for Regenerative Medicine, Faculty of Medicine Clínica Alemana-Universidad del Desarrollo, Santiago 7710162, Chile; paberrios@ug.uchile.cl (P.B.-C.); mquezadad@udd.cl (M.Q.); mezquer@udd.cl (M.E.); 2 Molecular and Clinical Pharmacology Program, Institute of Biomedical Sciences, Faculty of Medicine, Universidad de Chile, Santiago 8380453, Chile; equintanilla@med.uchile.cl (M.E.Q.); pmorales@uchile.cl (P.M.); mh_marschitz@uchile.cl (M.H.-M.); 3 Department of Neuroscience, Faculty of Medicine, Universidad de Chile, Santiago 8380453, Chile

**Keywords:** drug addiction, neuroinflammation, oxidative stress, treatment

## Abstract

Drug abuse is a major global health and economic problem. However, there are no pharmacological treatments to effectively reduce the compulsive use of most drugs of abuse. Despite exerting different mechanisms of action, all drugs of abuse promote the activation of the brain reward system, with lasting neurobiological consequences that potentiate subsequent consumption. Recent evidence shows that the brain displays marked oxidative stress and neuroinflammation following chronic drug consumption. Brain oxidative stress and neuroinflammation disrupt glutamate homeostasis by impairing synaptic and extra-synaptic glutamate transport, reducing GLT-1, and system X_c_^−^ activities respectively, which increases glutamatergic neurotransmission. This effect consolidates the relapse-promoting effect of drug-related cues, thus sustaining drug craving and subsequent drug consumption. Recently, promising results as experimental treatments to reduce drug consumption and relapse have been shown by (i) antioxidant and anti-inflammatory synthetic molecules whose effects reach the brain; (ii) natural biomolecules secreted by mesenchymal stem cells that excel in antioxidant and anti-inflammatory properties, delivered via non-invasive intranasal administration to animal models of drug abuse and (iii) potent anti-inflammatory microRNAs and anti-miRNAs which target the microglia and reduce neuroinflammation and drug craving. In this review, we address the neurobiological consequences of brain oxidative stress and neuroinflammation that follow the chronic consumption of most drugs of abuse, and the current and potential therapeutic effects of antioxidants and anti-inflammatory agents and biomolecules to reduce these drug-induced alterations and to prevent relapse.

## 1. Introduction

### 1.1. Drug Abuse and Current Treatments

Drug abuse is a major burden to society. Globally, 43% of the population 15 years and older are current alcohol drinkers [1], 19.2% are current smokers [2], and 5.5% are current illicit drug users, including cannabis [3]; many are multiple drug users [4,5]. Combined, legal and illegal drug use is estimated to cost over $700 billion annually only in the US [6], and is a major cause of preventable morbidity and mortality [7]. Despite such a heavy toll, there are insufficient pharmacological treatments to reduce the abuse of drugs. The U.S. Food and Drug Administration (FDA) has only approved medications for the treatment of alcohol [8], nicotine [9], and opioid abuse [10]; none for multiple drug use [5]. Treatments against the abuse of other drugs have been shown to be ineffective [11] or inconclusive [12]. The first line of treatment against alcohol abuse, naltrexone, and acamprosate, only moderately reduces alcohol drinking, as shown in a subset of studies [13]. Current therapies for nicotine abuse, varenicline, and bupropion, reduce smoking events, and increase abstinence [14], while effective for only short periods [15]. Opioid substitution therapy employs the replacement of the abused opioids with the longer-acting opioids methadone and buprenorphine, which reduces both craving and the risk of overdose [16,17]. However, opioid substitution needs to be highly regulated and maintains the brain changes induced by abused opioids [18,19]. In addition to low compliance, unsuccessful treatments are the result of self-perpetuating brain alterations caused by the abused substance, which control behavior and hinder the ability of patients to reduce their consumption and avoid relapse [20].

Rather than fully examining the mechanisms of drug abuse and dependence, this review addresses the pivots linking the brain changes that promote consumption and relapse with those resulting from drug-induced oxidative stress and neuroinflammation; how these alterations are exacerbated after drug consumption; and how chemical agents and biological molecules which blunt oxidative stress and inflammation could be used as treatments to restore drug-altered mechanisms, reduce drug consumption and prevent relapse.

### 1.2. Drugs of Abuse Activate the Brain Reward System

Drugs of abuse are reinforcing and promote chronic relapse. These drugs pharmacologically stimulate the brain reward system, causing a rise of dopamine synaptic levels that modulate the activation of medium spiny neurons in the nucleus accumbens (NAcc), at terminals from neurons that originate in the ventral tegmental area (VTA) [21,22]. The mechanisms by which abused substances increase dopamine levels are diverse, ranging from indirectly activating the dopaminergic neurons in the VTA, like opioids [23], to preventing dopamine from exiting the synapses in the NAcc, like cocaine [24,25]. A summary of the mechanisms by which abused substances promote the release of dopamine is showed in Table 1. 

The release of dopamine in the NAcc increases motivation for drug consumption, which is critical for the initial steps of drug use to further develop into a relapsing disorder [34,35,36]. After repeated consumption, the dopamine-dependent motivation progresses into a conditioned motivation that pairs drug consumption to environmental cues that predict drug availability. The transition to this learned behavior is governed by glutamatergic neurons from the prefrontal cortex, the hippocampus, and the amygdala, which stimulate the same medium spiny neurons innervated by the VTA [37,38,39]. Repeated drug consumption increases glutamatergic activity by impairing glutamate transport, altering glutamate synaptic and extrasynaptic levels defined as a change in glutamate homeostasis [37]. In addition, drug withdrawal promotes neuronal plasticity that enduringly potentiates glutamatergic activity, including a higher expression of glutamate AMPA receptors that allow Ca^2+^ influx, strengthening synaptic activity, which further reduces the brain ability to dissociate the environment pairing to drug consumption and overcome drug craving [37,38,40]. Recent evidence suggests that oxidative and inflammatory factors are critical contributors to the persistent alteration of glutamate homeostasis and contribute to the exacerbated glutamatergic activation of the NAcc (vide infra). Overall, the mechanisms that promote this enduring deviation of the NAcc function consolidate the association of drug consumption and environmental cues, promoting the chronic relapse to drug consumption. 

## 2. The Oxidative Stress and Inflammation Role in Drug Consumption

### 2.1. The Brain Oxidative Stress and Neuroinflammation Vicious Cycle

**Figure 1:**
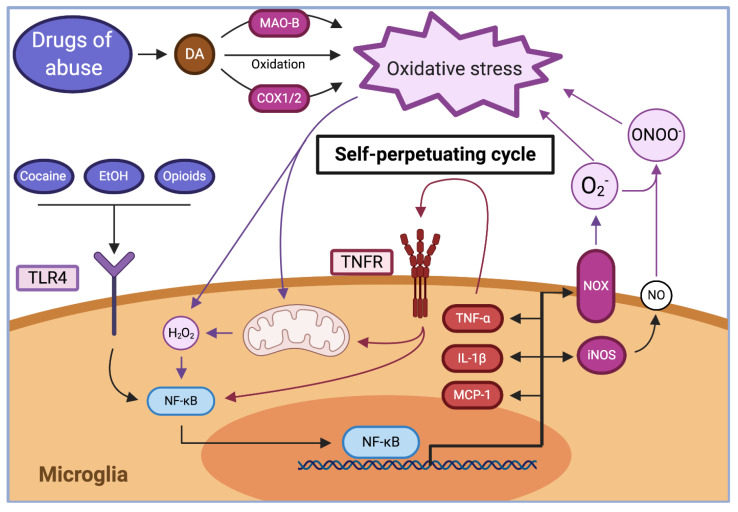
Drugs of abuse increase the levels of brain oxidative stress and trigger a self-perpetuating cycle that sustains neuroinflammation. Drugs of abuse promote the release of dopamine (DA), which is either rapidly spontaneously oxidized or metabolized via monoamine oxidase B (MAO-B) and cyclooxygenase 1/2 (COX1/2), generating superoxide ion and hydrogen peroxide, thus increasing oxidative stress. Oxidative stress generated by drugs of abuse can impair mitochondrial function, further increasing oxidative stress production. The rise in oxidative stress promotes the nuclear translocation and activation of NF-κB in microglia. Cocaine, ethanol, and opioids promote the activation of Toll-like receptor 4 (TLR4), which also activates microglial NF-κB. In the nucleus, NF-κB promotes the increase in the expression of the pro-oxidant enzymes NADPH-oxidase (NOX) and inducible nitric oxide synthase (iNOS), and of pro-inflammatory cytokines tumor necrosis factor α (TNF-α), interleukin 1β (IL-1β), and monocyte chemoattractant protein-1 (MCP-1). The increase of oxidative stress and pro-inflammatory cytokines promote the activation of microglia and astrocytes via the activation of NF-κB directly or via additional mitochondrial impairment, which further perpetuates the brain oxidative stress and inflammation cycle.

This review shows the existence of a drug-induced brain oxidative stress-neuroinflammation vicious cycle, which can start from either one of its components; and often by both. Most of the literature in the addiction field has dealt with drug-induced neuroinflammation (vide infra. The brain, an organ with specialized immune surveillance, is relatively isolated by the blood-brain barrier (BBB) from systemic immune cells and signals from the periphery [41,42]. However, the brain can mount its own inflammatory response against local pathogens or tissue damage, via the activation of astrocytes, the major keeper of brain homeostasis, and microglia, the local immune cells [43,44]. Both cell types produce cytokines and express pattern recognition receptors (i.e., Toll-like receptors, TLRs) that allow these to survey the environment and modulate a response [44,45]. Microglia respond to pro-inflammatory factors by changes in their phenotype and gene expression, increasing their levels of oxidant enzymes such as NADPH-oxidase (NOX) and inducible nitric oxide synthase (iNOS), generating reactive oxygen species (ROS) and reactive nitrogen species (RNS) [46] (Figure 1). 

The brain, comprising only 2% of body weight, utilizes 20% of all the oxygen consumed by the body. Neuronal activity is almost exclusively obtained from ATP generated in the mitochondria, rather than depending on glycolysis [47]. Mitochondria oxidizes O_2_ to H_2_O to produce ATP, but incomplete O_2_ oxidation generates O_2_^−^ and H_2_O_2_ that are rapidly controlled under normal conditions [48]. While heavy reliance on mitochondria allows neurons to fulfill their energy requirements, it leaves them vulnerable to excessive O_2_^−^ and H_2_O_2_ production in case of mitochondrial dysfunction as resulting from neuroinflammation [49]. In addition, neuron sensitivity is exacerbated by their low levels of antioxidant catalase and low levels of glutathione [50]. Similar to microglia, neurons express NOX and neuronal nitric oxide synthase (nNOS), generating signals that promote physiological synaptic transmission and plasticity [51]. However, excessive ROS signaling to microglia and astrocytes promote the generation of pro-inflammatory cytokines, mediated by the activation of NF-κB [52,53]. Pro-inflammatory cytokines in turn activate microglia and astrocytes, increasing the production of oxidative stress. Noteworthy, oxidative stress and inflammation further self-potentiate each other [54]. This self-perpetuating cycle could lead to a sustained inflammation that will be detrimental long after the initial trigger affected the brain (Figure 1). Indeed, increased levels of pro-inflammatory cytokines can be observed in the brain of mice up to 10 months after an acute systemic administration of inflammatory lipopolysaccharide (LPS) [55].

### 2.2. Drug Consumption Promotes Brain Oxidative Stress and Neuroinflammation

Studies in animals and humans have shown that a significant rise in peripheral and brain inflammation signals exists following the chronic intake of most drugs of abuse [56,57,58,59]. The activation of microglia and/or astrocytes has been shown in animal models of every drug studied including amphetamines [60,61], cocaine [62], ethanol [63,64,65,66], opioids [67], cannabinoids [68,69], and nicotine [65,66]. Interestingly, though the different drugs of abuse exert different mechanisms to promote the release of dopamine in nucleus accumbens, and motivate consumption (Table 1), they exert similar mechanisms to increase both brain oxidative stress and neuroinflammation. Specific mechanisms are discussed below.

#### 2.2.1. Dopamine Oxidation

Dopamine release per se may be sufficient to increase oxidative stress via its spontaneous and enzymatic oxidation (Table 2, Figure 1). Released dopamine leaves its protected environment inside low-pH synaptic vesicles to a neutral-pH synaptic environment where it can be spontaneously oxidized, forming dopamine quinones and superoxide radicals [70]. Both may initiate reaction chains if not efficiently cleared [71]. Dopamine quinones are not stable, they interact with cysteine residues in proteins forming adducts, which induces neurotoxicity in vitro [72], or cyclize forming aminochrome [70]. Dopamine oxidation can also be catalyzed by transition metals (like copper, which is present at synapses [73]) or enzymatically by cyclooxygenase 1 and 2 (COX1/2) [74,75], also forming dopamine quinones, and monoamine oxidase-B (MAO-B), which catalyzes dopamine deamination forming hydrogen peroxide [76,77] a molecule that can readily form hydroxyl radicals by the Fenton reaction. In addition, activated microglia can exacerbate dopamine oxidation via the release of reactive superoxide to the extracellular medium [71] while exposure of microglia to dopamine quinones promotes their activation [78]. Glutathione treatment, superoxide dismutase over-expression, and increasing the quinone oxidoreductase activity prevent the formation of dopamine quinone-protein adducts and the neurotoxicity induced by dopamine [72].

#### 2.2.2. Inhibition of System X_c_^−^ Activity

The system X_c_^−^ is a cystine-glutamate antiporter dimer composed of the catalytic subunit xCT and 4F2hc [79,80]. The system X_c_^−^ is expressed in astrocytes and microglia where it exerts a double function, it promotes the released glutamate to extrasynaptic space while cystine is taken up by the cell [81]. Intracellular cysteine is rapidly reduced to two molecules of cysteine that are used as a substrate for de novo synthesis of glutathione. Therefore, this protein simultaneously promotes glutathione synthesis, increasing the antioxidant protection against pro-oxidant drugs of abuse, and supports the glutamate homeostasis (discussed in Section 2.3) [81]. Reduced levels of activity of system X_c_^−^ has been shown in animal models of drug abuse, including cocaine [82,83], ethanol [84], and nicotine [85]. A mechanism for system X_c_^−^ activity inhibition may be drug-induced oxidative stress, as a molecular dynamics analysis show that the oxidation of a key cysteine residue of xCT blocks cystine access to the transporter [86]. In addition, the treatment of animals and drug abusers with the cysteine prodrug, N-acetylcysteine (NAC), which increases the activity of system X_c_^−^, reduces drug consumption as further discussed.

#### 2.2.3. Drug-Induced Mitochondrial Dysfunction

Mitochondrial generation of brain oxidative stress is a physiologically controlled process [48]. However, mitochondrial alterations have been observed in animal models of drug abuse. Specifically, alterations of brain mitochondrial DNA occur in animals after the administration of ethanol, amphetamines, cocaine, and morphine [87,88], which can alter mitochondrial electron flow and increase ROS production [89]. In the case of cocaine, the treatment of mitochondria isolated from rat brain reduced complex I activity [90], which increases O_2_^-^ production [91]. In addition, the presence of mitochondrial ROS may be necessary for the neuroinflammation caused by cocaine, since pre-treatment of mouse primary microglia with the mitochondrial ROS scavenger mitoTEMPO inhibited microglial activation and reduced the proinflammatory the rise in tumor necrosis factor-α (TNF-α), IL-1β, and IL-6 mRNA levels caused by cocaine treatment [92].

#### 2.2.4. Peripheral Inflammation Contributes to Neuroinflammation

The systemic routes of self-administration of drugs of abuse, such as oral intake or intravenous injection, indicate that many organs will be affected by their pro-oxidant and pro-inflammatory effects, in addition to the brain. Indeed, systemic cocaine administration increases the levels of pro-inflammatory cytokines in the colon and serum of mice [93] and cocaine users have higher levels of pro-inflammatory cytokines in serum than healthy controls [94,95]. This effect has been mostly studied for alcohol abuse, as ethanol is oxidized to acetaldehyde in the gut mucosal by bacterial alcohol dehydrogenase [96]. Acetaldehyde is a reactive molecule that forms stable adducts with proteins and DNA [97] and promotes the influx of gut-derived pro-inflammatory bacterial LPS to the peripheral circulation by disrupting the intestinal barrier in animals and alcoholic patients [57,96]. Leaked LPS increases the levels of serum TNF-α, after ethanol administration [98]. Increased levels of systemic pro-inflammatory cytokines, mainly TNF-α, can promote neuroinflammation by two mechanisms: they can directly activate brain TNF-α receptors, or activate BBB endothelial cells and pericytes, promoting their release of pro-inflammatory cytokines towards the brain [55,99]. The consequences of these mechanisms have been observed in animals and humans. Mice treated systemically with LPS increase their ethanol consumption [100], and in alcoholics, the levels of pro-inflammatory cytokines in plasma are positively correlated with a high alcohol craving score [101,102]. In addition, antibiotics treatment that reduces the intestinal bacterial load reduces brain cortex mRNA levels of the pro-inflammatory cytokines TNF-α, MCP-1, and IL-17, and the activation of microglia in the hippocampus induced by ethanol forced self-administration in mice [103] and markedly inhibited ethanol intake and relapse in rats bred for their high ethanol consumption [104]. 

#### 2.2.5. Activation of Toll-like Receptors

Some drugs of abuse are recognized as foreign molecules by the brain immune system pattern recognition receptors as if invaded by a foreign organism, mounting an inflammatory and oxidative response. Specifically, cocaine [105] and opioids [67,106,107] have been shown to be agonists of TLR4. Indeed, TLR4 activation by cocaine and opioids in microglia and astrocytes promotes their activation and the secretion of pro-inflammatory cytokines including TNF-α, IL-1β, and IL-6 [67,105,107,108]. In addition, ethanol also activates TLRs by promoting the release of HMGB1 (a TLR2 and TLR4 endogenous agonist) [109,110,111]. In vitro, cocaine and ethanol exposure to cultured microglia increases TLR4 protein levels [62,112]. The relevance of this mechanism for drug abuse has been shown as TLR4 antagonists block morphine conditioned place preference [113], heroin, and cocaine self-administration reinstatement [114,115] and reduce remifentanil self-administration in rats [113,114]. Treatment with HMGB1 neutralizing antibodies or TLR4 antagonists inhibits ethanol-induced increases of TNF-α, IL-1β, and MCP-1 mRNA in brain slices [110,111]. Similarly, TLR4 knockout mice do not show oxycodone conditioned place preference [113] and forced consumption of ethanol to TLR2 and TLR4 knockout mice fail to promote the increase of IL-1β and MCP-1 [63] or microglial activation [64]. Mice lacking CD14, an essential protein for TLR sensing function in microglia [116], or lacking TLR2 reduces their ethanol consumption [117,118].

### 2.3. Effect of Oxidative Stress and Inflammation on Glutamate Signaling

Glutamate homeostasis is strongly regulated by glial and neuronal mechanisms [119]. Most extracellular glutamate is not dependent on neuron depolarization [120], but the equilibrium of extrasynaptic efflux and uptake [80,121]. Astrocytes and microglia release glutamate to extrasynaptic space via the cystine-glutamate antiporter system X_c_^−^ [80], which releases glutamate into an extrasynaptic space [81] (the antioxidant effect of system X_c_^−^ by cystine internalization is discussed in Section 2.2). Among the different glutamate transporters, the astrocyte excitatory amino acid transporter 2 (EAAT2, also called GLT-1) accounts for ~90% of glial (synaptic) uptake in the brain [122] (reviewed by Danbolt [123]). GLT-1 is the most highly expressed glutamate transporter in the telencephalon and midbrain [124]. By the modification of system X_c_^−^ and GLT-1 activity, the extracellular levels of glutamate are altered after the administration of drugs of abuse (vide infra).

#### 2.3.1. Glutamate Transport Activity Is Impaired by Brain Oxidative Stress and Inflammation Components

Oxidative stress components (including ROS and RNS) can directly inhibit GLT-1 activity [125,126]. ROS inhibits glutamate transport, in part by promoting the cross-linking of cysteine residues [127,128] and by the formation of adducts of glutamate transporters with lipoperoxidation products like 4-hydroxynonenal and 4-hydroxyhexenal, which reduce GLT-1 activity [129]. Inhibition of glutamate transport by ROS is completely reversed by reducing agents in vitro [128,130,131,132,133]; while the inhibition induced by the RNS peroxynitrite (ONOO^−^) is less prone to reversal [127], possibly due to tyrosine nitration of GLT-1 [134]. Although less studied, system X_c_^−^ activity may also be inhibited by oxidative stress, as molecular dynamics studies show that oxidation of a key cysteine residue of xCT impairs its activity [86].

Activation of rat microglia by LPS treatment has been shown to generate oxidative stress, mainly releasing ONOO^−^, and to promote glutamate uptake inhibition [135]. In vitro, human and rat astrocytes treated with the pro-inflammatory cytokines TNF-α and IL-1β for 30 min to 7 days reduce their glutamate uptake [136,137,138,139,140,141], an effect that is abolished by pre-treatment with NOS inhibitors [138,140] and replicated by treatment with nitric oxide donors [138]. Pro-inflammatory cytokines reduce GLT-1 mRNA and protein levels, further blunting glutamate uptake [142,143]. 

#### 2.3.2. Drugs of Abuse Modify the Extracellular Levels of Glutamate

**Figure 2:**
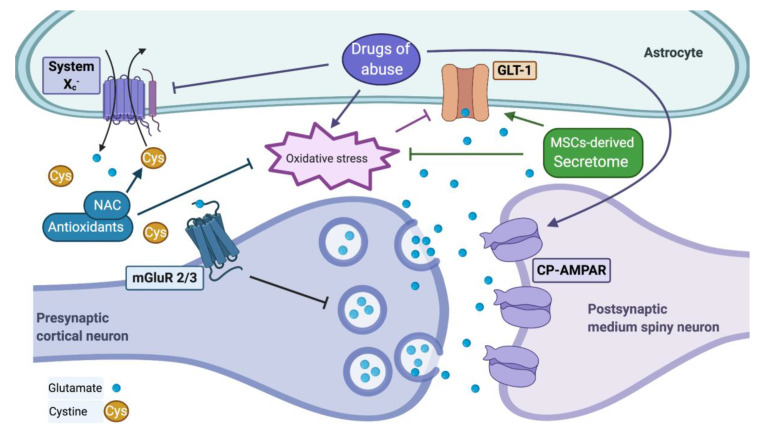
The tripartite synapse and glutamate homeostasis. When glutamate homeostasis is maintained, GLT-1 removes glutamate from the synaptic cleft, and the glial system X_c_^−^ releases glutamate to the extrasynaptic location, which activates the inhibitory metabotropic glutamate receptors 2 and 3 (mGluR2/3) which inhibits synaptic glutamate release. Drugs of abuse alter glutamate homeostasis by increasing the levels of oxidative stress, which impair GLT-1 activity and reduce the activity of system X_c_^−^. Both effects potentiate cued-induced increases in post-synaptic glutamate tone. Drugs of abuse also strengthen glutamatergic transmission by promoting the recruitment of Ca^2+^ permeable AMPA receptors (CP-AMPAR). Antioxidants like N-acetylcysteine (NAC) normalize glutamate homeostasis by increasing the levels of cystine, activating both the system X_c_^−^ and mGluR2/3, also increasing GSH and reducing oxidative stress, which recovers GLT-1 activity. Administration of secretome derived from mesenchymal stem cells (MSCs) also reduces drug consumption by reducing oxidative stress and recovering GLT-1 activity.

The net effect of drugs of abuse on the levels of extracellular glutamate will depend on the degree of impairment of the system X_c_^−^, which normally releases glutamate from astrocytes and microglia into the extracellular space [37]. Normalizing the glutamate homeostasis allows the brain to break away from the drug-cue learned association that promotes relapse [83,144,145]. Rat models of cocaine addiction show low extracellular glutamate levels in the NAcc after a cocaine self-administration extinction period [82], and restoring X_c_^−^ activity either by the administration of cystine directly into the NAcc or by systemic administration of the antioxidant N-acetylcysteine (NAC, a cysteine pro-drug) normalizes extracellular glutamate levels and reduces cocaine-cued seeking behavior [82]. The increased X_c_^−^ activity normalizes the release of glutamate into the extra-synaptic compartment, which activates the inhibitory metabotropic glutamate receptors 2 and 3 (mGluR2/3) located in the pre-synaptic neuron. Indeed, direct activation of mGluR2/3 reduces the release of synaptic glutamate, further contributing to the normalization of glutamate homeostasis [146] (Figure 2). Interestingly, the activation of mGluR2/3 by NAC also restores the NAcc ability to recover neuronal synaptic adaptations. Consequently, the antagonists of mGluR2/3 prevent the rescuing effect of cocaine-seeking behavior and relapse to ethanol consumption by NAC [144,147,148].

Methamphetamine self-administration also reduced extracellular glutamate levels in the NAcc and prefrontal cortex of rats after an extinction period [149]. Under a different experimental paradigm, the opposite effect was observed, as a three-week withdrawal of methamphetamine self-administration increased the levels of extracellular glutamate in the NAcc [150], suggesting that in this model the glutamate transport by GLT-1 is more impaired than the X_c_^−^ -mediated glutamate extrasynaptic release. Increased glutamate levels were also observed in the NAcc of mice exposed to alternating forced ethanol administration with self-administration, after 18 h of abstinence [151,152]. In these experiments, direct administration of a mGluR2/3 agonist into the NAcc did significantly reduce ethanol consumption [151]. In rats, ethanol self-administration increased extracellular glutamate levels in the NAcc and VTA [153]. Systemic NAC administration after a period of abstinence from ethanol or nicotine self-administration significantly reduced their respective substance reinstatement, compared to rats administered with saline [65]. In rodent models, the relevance of synaptic (GLT-1) glutamate transport on ethanol and nicotine self-administration was additionally demonstrated by the intranasal administration of the antioxidant and anti-inflammatory secretome derived from mesenchymal stem cells (MSCs), which significantly inhibited the consumption of both drugs, an effect that was fully prevented by brain GLT-1 knockdown [66]. Overall, evidence suggests that glutamate transport by GLT-1 and glutamate glial release by system X_c_^−^ are impaired in drug abuse models, regardless of the net effect on the brain extracellular glutamate levels.

#### 2.3.3. The Recovery of GLT-1 and System X_c_^−^ Activities Inhibits Drug Seeking and Reinstatement

Several pharmacological treatments have been developed to study the effect of the recovery of GLT-1 and system X_c_^−^ activities. Screening more than 1000 FDA approved drugs, Rothstein et al. [154] showed that β-lactam antibiotics were potent inducers of GLT-1 expression, significantly raising their mRNA and protein levels. Since then, ceftriaxone has been the most commonly used β-lactam to increase GLT-1 levels, which was subsequently found to also promote the expression of xCT and increase system X_c_^−^ activity [155]. Ceftriaxone has been shown to reduce the reinstatement of drug paired behavior and seeking of cocaine [156], amphetamine [157], ethanol (including direct inhibition of consumption) [158,159,160], opioids [161], and nicotine [162], showing the broad-spectrum of the recovering of glutamate homeostasis in the treatment of drug addiction. 

Despite the promising preclinical results indicated above, the applicability of ceftriaxone as a treatment for drug abuse in humans is limited. As an antibiotic, its chronic use could induce bacterial resistance and it is prone to generate gastrointestinal secondary effects. In addition, ceftriaxone parenteral administration would reduce the compliance of drug consumers. Thus, alternative inducers of GLT-1 and system X_c_^−^ activities are necessary to translate the favorable results in animal models to clinical trials.

## 3. Evidence for the Therapeutic Potential of Antioxidant and Anti-inflammatory Agents for the Treatment of Chronic Drug Abuse

### 3.1. N-Acetylcysteine (NAC)

Besides being used as a pharmacological tool to increase the activity of glutamate transporters system X_c_^−^ and GLT-1, as discussed in Section 2.3.1, preclinical and clinical evidence shows the antioxidant potential of NAC as a treatment for drug abuse. NAC antioxidant activity is explained by its direct reducing action of disulfide bonds and by being a precursor of the potent antioxidant glutathione [163]. Thus, NAC treatment successfully normalizes the rat brain levels of oxidative stress and inflammation raised by exposure to drugs of abuse like ethanol, nicotine [65,164], and methamphetamine [165], which correlated with a reduction of oral intake of ethanol and nicotine after months of ad libitum consumption in NAC-treated rats [65,166]. The reduction of ethanol and nicotine intake is more pronounced if orally administered NAC is given repeatedly during drug intake [65,166] or intraperitoneally as a bolus at the end of a period of abstinence [167]. As expected, the reduction of rat ethanol consumption was enhanced if NAC was given as a co-treatment with the anti-inflammatory drug aspirin [166], which also reduces the levels of inflammatory factors in the brain (see below). NAC treatment also prevents the reinstatement of bar-pressing behavior conditioned by opioids, cocaine, and cannabinoids after an extinction period [82,168,169,170]. Given these broad-spectrum encouraging results of NAC, in addition, to be currently an FDA-approved and well-tolerated drug for the treatment of acetaminophen poisoning [171], clinical trials have been performed to determine whether NAC could reduce drug intake in humans. A recent meta-analysis of 7 articles—representing the treatment of methamphetamine, cannabis, cocaine, and nicotine addiction—showed that NAC (prescribed to be administered twice-daily) moderately but significantly reduces craving symptoms compared to placebo [172]. Low compliance with medication intake was however noted in some studies, which is compounded by the short biological half-life of NAC of 6 h [173]. Longer-acting means of reducing oxidative stress and neuroinflammation would be preferable (vide infra). Noteworthy, the trials found that NAC is effective in preventing relapse, rather than reducing ongoing consumption [174,175], which is consistent with its proposed mechanism of action, that is the restoration of glutamate homeostasis and the dissociation of drug and environmental stimuli pairing (Section 1.2). Additionally, given the drug-induced development of a self-perpetuation oxidative mechanism, an antioxidant drug will be more effective if the use of very drug that generated oxidative stress is halted.

### 3.2. Ibudilast

The anti-inflammatory drug, ibudilast, is a well-tolerated FDA-approved treatment for respiratory asthma, due to its inhibition of smooth muscle contractibility [176,177]. The Ibudilast anti-inflammatory effect is the result of the inhibition of cyclic nucleotide phosphodiesterases (PDEs) [178,179]. In animals administered with LPS, ibudilast treatment reduces TNF-α release by microglia and astrocytes [180]; additionally in vitro addition of ibudilast to LPS-activated microglia reduces ROS and nitric oxide release and the mRNA levels of the pro-inflammatory cytokines IL-1β, IL-6, and TNF-α, while increasing the mRNA level of the anti-inflammatory cytokine IL-10 [181]. The activation of microglia and astrocytes induced by morphine administration in rats is also reduced by ibudilast treatment, an effect that was correlated with reduced mRNA levels of IL-1β in the prefrontal cortex [182]. In addition, ibudilast significantly reduced ethanol intake in rats and mice [183], and the methamphetamine-primed bar-pressing reinstatement was previously conditioned by methamphetamine self-administration [184]. In humans, ibudilast has a biological half-life of 19 h, which allows constant plasma levels [185]. Patients presenting an alcohol use disorder reduced their craving for alcohol after ibudilast treatment [186], and methamphetamine users reduced the self-report of subjective rewarding effects of the drug after ibudilast treatment compared to placebo-treated users [187]. 

### 3.3. Non-Steroidal Anti-Inflammatory Drugs (NSAIDs)

NSAIDs are non-specific inhibitors of cyclooxygenases 1 and 2 (COX1 and 2). The anti-inflammatory activity of NSAIDs occurs due to the inhibition of pro-inflammatory prostaglandin synthesis, by COX1 or COX2 [188]. Recent evidence showed that NSAIDs could be beneficial for the treatment of drug addiction. Mice treated with the COX1 and COX2 inhibitor indomethacin prevented the activation of microglia and astrocytes, as well as the rise of the pro-inflammatory cytokine TNF-α induced by methamphetamine administration [189]. Similarly, rats treated with amphetamine that received the COX2 inhibitor celecoxib did not show the rise in TNF-α levels in the striatum that was observed in rats treated with amphetamine alone [190]. In addition, the administration to alcohol-dependent animals of aspirin alone, or in combination with NAC significantly reduced the voluntary ethanol consumption of treated animals [166]. Aspirin by itself administered at doses used in humans, inhibited oxidative stress in the hippocampus of chronically ethanol ingesting animals [166]. The treatments also reduced the brain oxidative stress, observed as the ratio of oxidized glutathione/reduced glutathione in the hippocampus, and prevented the activation of astrocytes and microglia [166]. Interestingly, aspirin treatment promotes additional anti-inflammatory mechanisms compared to other NSAIDs, as COX2 acetylated by aspirin can metabolize arachidonic acid to induce the generation of aspirin-induced lipoxins, with potent anti-inflammatory activity [191]. Recently, lipoxin administration was found to significantly inhibit chronic ethanol intake in rats and the administration of a lipoxin antagonist prevented the effect of aspirin in reducing ethanol intake [148], again showing a clear association between dug intake and inflammation.

## 4. Biomolecules as Potential Treatments of Drug Abuse

### 4.1. Mesenchymal Stem Cells and Their Products

The administration of mesenchymal stem cells (MSCs) has emerged as a promising therapeutic strategy for brain neurodegenerative and inflammatory diseases due to their pleiotropic mechanisms of actions that include cell replacement, promotion of survival via paracrine signaling, modulation of inflammation, and reduction of oxidative damage [192]. Among the different types of adult stem cells, MSCs are one of the most employed in pre-clinical studies since these: (i) can be easily isolated from different tissues (e.g., fat depots, bone marrow, cord blood, menstrual blood) and expanded in vitro; (ii) exhibit great proliferative potential [193]; (iii) produce high levels of antioxidant enzymes including superoxide dismutase, catalase, and glutathione peroxidase; thus being able to reduce oxidative stress [194]; (iv) have potent immunomodulatory properties mediated by the secretion of anti-inflammatory molecules and the inhibition of microglia and astrocyte activation [195,196,197] (thus having a dual impact blunting the agents that promote the oxidative stress-neuroinflammation potentiation), and (v) have a good safety profile [198]. Importantly, MSCs show low immunogenicity [199], allowing allogeneic transplantation without immunosuppression. In addition, preclinical studies show that xenogeneic approaches (i.e., human MSCs transplanted into animal models) are effective in immunomodulation, recovery of function, and reduction of brain disease progression [200,201], which should allow the evaluation of the efficacy of human MSCs in animal models of several pathologies. Importantly, the administration of MSCs has successfully reduced brain oxidative stress and inflammation in animal models of oxidative and inflammatory diseases, including stroke, cerebral ischemia, traumatic brain injury and multiple sclerosis [196,202,203,204].

#### 4.1.1. MSCs Intra-Cranial Administration Reduces Ethanol Intake and Relapse in Rats

Due to their potent antioxidant and anti-inflammatory properties MSCs appear as highly attractive candidates for the treatment of addictions. In significant proof-of-concept studies, our research group demonstrated that a single intra-cerebroventricular administration of rat bone marrow-derived or adipose tissue-derived MSCs [205], or human adipose tissue-derived MSCs [206] to rats that had consumed ethanol chronically reduced their ethanol intake by 75% and inhibited alcohol relapse up to 85%. Such an effect was also achieved following the intravenous administration of spheroids derived from human MSCs to rats after chronic ethanol intake [207] (spheroids are generated by culturing MSCs in hanging droplets to reduce their size and increase their production of anti-inflammatory factors). Of note is the long-acting effect of spheroids; in high drinker rats, a single intravenous administration of MSC spheroids inhibited (>75%) chronic ethanol intake for 3 weeks, and subsequently, by the fifth week it also reduced the post-deprivation relapse binge-like drinking by 75% compared to vehicle-treated rats [207]. The therapeutic effects of MSCs administration were correlated with a significant reduction of brain oxidative stress and neuroinflammation and with an increase of GLT-1 protein levels in the NAcc and prefrontal cortex [206,207]. 

#### 4.1.2. MSCs-Derived Secretome: A Safer Product Recapitulates the Effect of Living MSCs Administration

Initially, MSCs were thought to exert their therapeutic effects due to their multipotent differentiation potential and by direct cell-to-cell interactions [208]. However, presently the most accepted therapeutic mechanism associated with MSCs action is the generation of a pro-regenerative microenvironment through the paracrine secretion of different bioactive molecules [209]. Thus, MSCs have been described as the “injury drugstore” [210]. Accordingly, several researchers have demonstrated in different animal models that the administration of secretome produced by in vitro cultured MSCs can recapitulate the therapeutic effect obtained following the administration of living MSCs [211,212]. Compared with the use of live MSCs, the MSC-derived secretome has several advantages including (i) higher biosafety, reducing the risk of thrombosis or tumor generation; (ii) ready availability, as secretome can be lyophilized favoring it storage; (iii) greater reproducibility of therapeutic effect since it composition can be defined and standardized; (iv) ease of administration since due to the small size of its components, the secretome can be non-invasively administered by the nasal route to reach the brain; and (v) the administration can be repeated over time, as it does not generate an immunological rejection. Additionally, the composition of the MSC secretome can be modified by subjecting these cells to an in vitro preconditioning stimulus. This leads to the secretion of an appropriate combination of bioactive molecules specific for a determined pathology [213]. In this sense, our research group and others have reported that the in vitro preconditioning of MSCs with pro-inflammatory cytokines induces the secretion of high levels of anti-inflammatory and antioxidant molecules [206,213,214]. The secretome derived from MSCs is made of both: (a) soluble bioactive molecules and (b) exosomes: small nanovesicles containing several cell components including proteins and microRNAs (miRNAs). Both fractions have been associated with the paracrine effects of MSCs [215]. 

#### 4.1.3. MSCs-Derived Secretome Administration Reduces Drug Consumption and Relapse in Animal Models

The secretome derived from MSCs has been used to treat drug consumption and relapse in animal models of drug addiction. Importantly, MSCs secrete catalase and superoxide dismutase and promote the up-regulation of glutathione peroxidase in treated tissues [214], which potentially prevents the oxidation of redox-sensitive glutamate transporters that promote consumption and relapse (see Section 2.3). Indeed, using an animal model of alcohol addiction, we demonstrated that the non-invasive intranasal administration of exosomes derived from preconditioned human MSCs (hMSCs) efficiently reached the brain and partially reproduced the relapse prevention effects observed after the administration of live MSCs [216], while the intranasal administration of the complete secretome (exosomes plus soluble molecules) derived from preconditioned hMSCs fully recapitulated the therapeutic effects of MSC administration including a 90% reduction of chronic alcohol intake, complete prevention of alcohol relapse, a full reversion of hippocampal oxidative stress and inflammation, and an increase of GLT-1 levels in NAcc [66]. Furthermore, the therapeutic effects could be maintained by the administration of additional secretome at weekly intervals [66]. The anti-addictive effects of MSC-derived secretome have also been shown in a nicotine abuse model. In animals that self-administered oral nicotine, the intranasal administration of secretome greatly reduced nicotine-induced brain oxidative stress and inflammation, and lead to a marked reduction of chronic nicotine self-administration and complete prevention of nicotine relapse [66]. These results show that the therapeutic-like effects of MSCs secretome treatment are not restricted to alcohol abuse. In both animal models of alcohol and nicotine abuse, the intracerebral administration of an antisense oligonucleotide directed against GLT-1 fully inhibited the secretome effects, strongly suggesting that the anti-addictive effect is mediated by the ability of the secretome to increase GLT-1 activity and levels, possibly due to the prevention of GLT-1 oxidation [66]. 

### 4.2. MicroRNA

MicroRNA (miRNA) are biologically synthesized molecules that regulate several processes by modulating multiple mRNA targets. These non-coding RNA molecules of ~22 nucleotides silence mRNA both via reducing the levels of their target mRNA and repressing translation [217]. MiRNAs are transcribed as longer sequences that are processed to be included in an RNA-induced silencing complex (RISC) [217,218]. RISC targets multiple mRNAs with a complementary sequence at the 3′ untranslated region (3′-UTR) and obstructs translation by either cleaving the mRNA target if the sequence pairing is complete or by destabilization and decapping if the pairing is imperfect [219,220]. The miRNA down-regulation of multiple targets can have a synergistic effect that steers the cell to act in a specific direction [221], or just fine-tunes gene expression by acting in conjunction with other regulators [217]. These characteristics indicate that miRNAs could be used as therapeutic tools for the modulation of specific targets, while the development of strategies for their delivery constitutes an ongoing challenge [222,223].

#### 4.2.1. MicroRNA Expressed After Pro-inflammatory or Anti-inflammatory Signals in the Brain

Some miRNAs are known to modulate the expression of molecules related to the oxidative stress response [224] or of inflammatory factors [225]. In the brain, microglial and astrocyte expression of miR-155 and miR-146 increases after the activation of TLR2 or TLR4 [226,227,228]. MiR-155 is the most up-regulated miRNA after microglia activation in vivo and it controls the inflammatory activity of the cells. MiR-155 inhibits the translation of anti-inflammatory proteins, including the suppressor of cytokine signaling-1 (SOCS-1), thus promoting the expression of iNOS and pro-inflammatory cytokines [229,230]. Conversely, inhibition of miR-155 reduces astrocyte inflammatory gene expression [231,232]. On the other hand, miR-146 is induced by NF-κB and has been shown to be anti-inflammatory. Indeed, overexpression of miR-146 inhibits iNOS and IL-1β expression via inhibition of interleukin 1 receptor-associated kinase 1 (IRAK1) and TNF receptor-associated factor 6 (TRAF6), suggesting a role on toning down microglial activation [233].

Additionally, the anti-inflammatory miR-124 is the most abundant miRNA in the brain, accounting for 25 to 48% of total miRNA in mouse cortex, midbrain, and cerebellum [234]. The expression of miR-124 prompts microglia to remain in a non-activated state and is down-regulated if microglia are exposed to pro-inflammatory stimuli [228,235]. In resting microglia, miR-124 activity directly inhibits the translation of the transcription factor CCAAT/enhancer-binding protein-α (CEBPA) [235]; being a promoter of differentiation of monocytic cells [236] that inhibits the microglial activation markers CD11b and CD45 [235]. 

#### 4.2.2. Pro- and Anti-Inflammatory miRNAs in Neurodegenerative Diseases. The case of miR-155, miR-146, and miR-124

The relevance of miRNA control of brain oxidative stress and neuroinflammation has been shown in neurodegenerative diseases [237]. A literature review of almost 1,000 articles showed that in neurodegenerative diseases the most consistently upregulated miRNAs include miR-155 and miR-146, while the most consistently down-regulated miRNAs include miR-124 [238]. Consistent with their discussed pro- and anti-inflammatory effects, inhibiting the expression of miR-155 or increasing the expression of miR146 and miR124 in animal models of neurodegenerative diseases is beneficial. For example, the intracerebroventricular administration of anti-miR-155 reduces microglial activation, brain oxidative stress, and inflammation markers, and increases survival in experimental models of amyotrophic lateral sclerosis [229] and traumatic brain injury [239]. Similarly, the intracerebroventricular administration of mice with miR-124 complexed in liposomes significantly reduced the amount of activated microglia compared to animals treated with control miRNA, in a model of autoimmune encephalomyelitis [235].

#### 4.2.3. The potential of Pro- and Anti-Inflammatory miRNA in the Modulation of Drug Abuse

##### MiR-155

**Figure 3:**
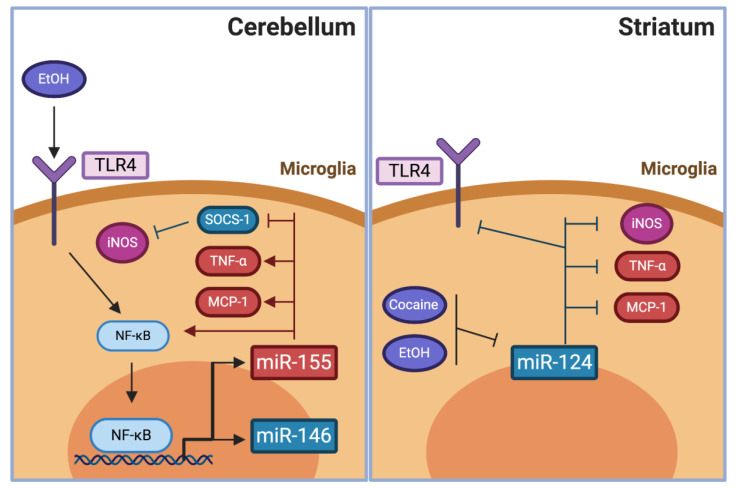
The effect of pro- and anti-inflammatory miRNA modulation in animal models of drug addiction. Ethanol consumption increases the levels of miR-155 and miR-146 in the cerebellum. Pro-inflammatory miR-155 expression promotes increases levels of pro-inflammatory cytokines and NF-κB activity and inhibits anti-inflammatory proteins like the suppressor of cytokine signaling (SOCS-1). Anti-inflammatory miR-124 microglial expression reduces pro-inflammatory cytokines, iNOS, and TLR4 levels; the effect that is inhibited in the striatum of animal models of cocaine or ethanol consumption.

Recent evidence showed that miR-155 promotes brain inflammation in animal models of ethanol abuse. Rats and mice increased their levels of cerebellar miR-155 after chronic ethanol consumption [240,241]. In mice, the rise of miR-155 after ethanol consumption occurred via TLR4 activation, since TLR4 knockout mice did not increase their miR-155 levels [241]. In addition, miR-155 knockout mice did not show the increases of cerebellar TNF-α and monocyte chemoattractant protein-1 (MCP-1) protein and mRNA levels, or NF-κB activity after ethanol consumption observed in wild-type animals [241] (Figure 3). The miR-155 expression also increased TNF-α peripheral levels, as seen in a mouse model of alcoholic liver disease [242]. The surge of peripheral inflammation is thought to contribute to the mechanism by which ethanol promotes brain oxidative stress and neuroinflammation (Table 2), which as discussed above promotes chronic alcohol intake and relapse in animal models and correlates with craving in abstinent alcohol use disorder patients. 

##### MiR-146

Similar to what has been observed in animal models of neurodegenerative diseases (see above), the levels of the anti-inflammatory miR-146 are increased in a rat model of ethanol consumption [240]. In addition, an association of miR-146 with human alcohol consumption has been found. In a microarray study, miR-146 was shown to be up-regulated in the frontal cortex of alcoholics compared to healthy controls [243]. In addition, the rs2910164 genetic variant of miR-146 was linked to patients with alcohol use disorders, though it is not known whether this variant expresses an altered miR-146 activity [244].

##### MiR-124

Reduced levels of anti-inflammatory miR-124 have been shown in models of drug abuse. In vitro, cocaine administration to a mouse microglial cell line reduced their miR-124 levels and increased their mRNA levels of TNF-α, iNOS, and MCP-1 [62,245] (Figure 3). Effects that were prevented by the overexpression of miR-124 [62]. In vivo, reduced levels of miR-124 were found in the hippocampus of rats treated systemically with cocaine for 15 days [246], and also in whole brain homogenates and purified microglia of mice treated with cocaine for 7 days [245]. In addition, miR-124 levels were lowered in the dorsolateral striatum of rats after 15 days of ethanol consumption [247]. The effect of modifying miR-124 levels in drug consumption has also been studied; the administration of a lentiviral vector (LV) coding for miR-124 into the striatum reduced the activation of microglia, the increases in TLR4 levels, and the increase in locomotor activity elicited by cocaine administration in mice [62]. Similar results were observed in rats using the conditioned place preference paradigm. In these experiments, the bilateral administration of an LV coding for miR-124 into the dorsolateral striatum reduced the place preference conditioning by cocaine administration compared with controls, while place conditioning was increased in rats which miR-124 levels were lowered further by the administration of a silencing LV prior to the cocaine administrations [248].

## 5. Conclusions

In recent years, common brain mechanisms that promote drug consumption and relapse have been uncovered. The enduring pairing of drug consumption and drug-associated cues that predict its availability is amplified by altered glutamate homeostasis induced by brain oxidative stress and neuroinflammation. A point made in this review is that no longer oxidative stress and neuroinflammation can be seen as separate; the literature strongly indicates that not only both are associated and potentiate each other but coexist in a vicious-like self-perpetuating cycle. Indeed, evidence that drugs of abuse increase both brain oxidative stress and neuroinflammation has been documented, which correlates with the impairment of glutamatergic homeostasis via the inactivation of the glial and redox-sensitive glutamate transporters GLT-1 and the system X_c_^-^. Thus, this review assessed the use of present and potential antioxidants or anti-inflammatory treatments for drug addiction. The consumption of different drugs of abuse and the relapse after abstinence in several preclinical models of drug addiction have been successfully inhibited after the treatment with the antioxidant NAC and anti-inflammatory molecules like ibudilast and aspirin. Potential new treatments such as those afforded by bioproducts released by mesenchymal stem cells or by modulating the levels of miRNA that target several oxidant and pro-inflammatory mechanisms have been proposed. These pre-clinically tested agents have shown marked reductions of consumption and relapse in animal models of ethanol, nicotine, and cocaine abuse. These new approaches may constitute useful treatments against the abuse of multiple drugs while requiring additional pre-clinical studies on different models of both drug consumption and withdrawal intake to ascertain their broad-spectrum potential.

## Figures and Tables

**Table 1:** Mechanisms by which drugs of abuse increase the dopamine levels in the nucleus accumbens.

Drug	Mechanism
Amphetamines	Substrates of the dopamine transporter. Promote the efflux of dopamine from cytosolic vesicles into the synaptic cleft [26].
Cocaine	Blockage of the dopamine transporter, increasing the levels of dopamine in the synaptic cleft [24,25].
Opioids	Activate µ-opioid receptors on GABAergic interneurons in the ventral tegmental area inhibiting them, which in turn disinhibits dopaminergic neurons that release dopamine in the nucleus accumbens [23].
Ethanol	Acts as a pro-drug through its first metabolite acetaldehyde, which reacts with dopamine to form the tetrahydroisoquinoline adduct salsolinol [27], likely an agonist of the µ-opioid receptor [28,29]. Salsolinol activates opioid receptors on GABAergic interneurons in the ventral tegmental area inhibiting them, which in turn disinhibits dopaminergic neurons that release dopamine in the nucleus accumbens [30].
Cannabinoids	Agonists of CB1 and CB2 receptors. Activate CB1 receptors on GABAergic interneurons in the ventral tegmental area, which in turn disinhibits dopaminergic neurons that release dopamine in the nucleus accumbens [31].
Nicotine	Activate α_4_β_2_ or α_6_β_2_ nicotinic acetylcholine receptors in mesolimbic dopaminergic neurons [32], which promotes the release of dopamine in the nucleus accumbens [33].

**Table 2:** Mechanisms by which drugs of abuse promote an increase in brain oxidative stress and inflammation.

Mechanism	Drugs Involved	References
Oxidation of dopamine	Every drug that increases dopamine levels	[70,71,78]
Inhibition of system X_c_^−^	Cocaine, ethanol, and nicotine	[81,82,83,84,85]
Drug-induced mitochondrial dysfunction	Ethanol, amphetamines, cocaine, morphine	[87,88,92]
Peripheral inflammation contributes to neuroinflammation	Ethanol, cocaine	[57,93,96]
Activation of Toll-like receptors	Cocaine, opioids, ethanol	[105,107,111]

## References

[1] WHO (2019). Global Status Report on Alcohol and Health 2018.

[2] WHO (2019). WHO Report on the Global Tobacco Epidemic 2019: Offer Help to Quit Tobacco Use.

[3] UNODC (2019). World Drug Report 2019.

[4] Bhalla I.P., Stefanovics E.A., Rosenheck R.A. (2017). Clinical epidemiology of single versus multiple substance use disorders: Polysubstance use disorder. Med. Care.

[5] Van Skike C., Maggio S., Reynolds A., Casey E., Bardo M., Dwoskin L., Prendergast M., Nixon K. (2016). Critical needs in drug discovery for cessation of alcohol and nicotine polysubstance abuse. Progress Neuro Psychopharmacol. Biol. Psychiatr..

[6] NIDA Trends & Statistics. https://www.drugabuse.gov/related-topics/trends-statistics.

[7] Peacock A., Leung J., Larney S., Colledge S., Hickman M., Rehm J., Giovino G.A., West R., Hall W., Griffiths P. (2018). Global statistics on alcohol, tobacco and illicit drug use: 2017 status report. Addiction.

[8] Soyka M., Müller C.A. (2017). Pharmacotherapy of alcoholism–an update on approved and off-label medications. Exp. Opin. Pharmacother..

[9] Gómez-Coronado N., Walker A.J., Berk M., Dodd S. (2018). Current and emerging pharmacotherapies for cessation of tobacco smoking. Pharmacother. J. Hum. Pharmacol. Drug Ther..

[10] FDA Information about Medication-Assisted Treatment (Mat). https://www.fda.gov/drugs/information-drug-class/information-about-medication-assisted-treatment-mat.

[11] Siefried K.J., Acheson L.S., Lintzeris N., Ezard N. (2020). Pharmacological treatment of methamphetamine/amphetamine dependence: A systematic review. CNS Drugs.

[12] Prince V., Bowling K.C. (2018). Topiramate in the treatment of cocaine use disorder. Bull. Am. Soc. Hosp. Pharm..

[13] Jonas D.E., Amick H.R., Feltner C., Bobashev G., Thomas K., Wines R., Kim M.M., Shanahan E., Gass C.E., Rowe C.J. (2014). Pharmacotherapy for adults with alcohol use disorders in outpatient settings: A systematic review and meta-analysis. JAMA.

[14] Jordan C.J., Xi Z.-X. (2018). Discovery and development of varenicline for smoking cessation. Exp. Opin. Drug Discov..

[15] Ebbert J.O., Croghan I.T., Sood A., Schroeder D.R., Hays J.T., Hurt R.D. (2009). Varenicline and bupropion sustained-release combination therapy for smoking cessation. Nicotine Tob. Res..

[16] Dahan A. (2006). Opioid-induced respiratory effects: New data on buprenorphine. Palliat. Med..

[17] Koehl J.L., Zimmerman D.E., Bridgeman P.J. (2019). Medications for management of opioid use disorder. Am. J. Health Syst. Pharm..

[18] Cunningham C.O., Kunins H.V., Roose R.J., Elam R.T., Sohler N.L. (2007). Barriers to obtaining waivers to prescribe buprenorphine for opioid addiction treatment among hiv physicians. J. Gen. Intern. Med..

[19] Noble F., Marie N. (2019). Management of opioid addiction with opioid substitution treatments: Beyond methadone and buprenorphine. Front. Psychiatr..

[20] Volkow N.D., Koob G.F., McLellan A.T. (2016). Neurobiologic advances from the brain disease model of addiction. N. Eng. J. Med..

[21] Di Chiara G., Imperato A. (1988). Drugs abused by humans preferentially increase synaptic dopamine concentrations in the mesolimbic system of freely moving rats. Proc. Natl. Acad. Sci. USA.

[22] Ikemoto S. (2007). Dopamine reward circuitry: Two projection systems from the ventral midbrain to the nucleus accumbens–olfactory tubercle complex. Brain Res. Rev..

[23] Johnson S.W., North R.A. (1992). Opioids excite dopamine neurons by hyperpolarization of local interneurons. J. Neurosci..

[24] Sora I., Hall F.S., Andrews A.M., Itokawa M., Li X.-F., Wei H.-B., Wichems C., Lesch K.-P., Murphy D.L., Uhl G.R. (2001). Molecular mechanisms of cocaine reward: Combined dopamine and serotonin transporter knockouts eliminate cocaine place preference. Proc. Natl. Acad. Sci. USA.

[25] Budygin E.A., John C.E., Mateo Y., Jones S.R. (2002). Lack of cocaine effect on dopamine clearance in the core and shell of the nucleus accumbens of dopamine transporter knock-out mice. J. Neurosci..

[26] Jones S.R., Gainetdinov R.R., Wightman R.M., Caron M.G. (1998). Mechanisms of amphetamine action revealed in mice lacking the dopamine transporter. J. Neurosci..

[27] Israel Y., Quintanilla M.E., Karahanian E., Rivera-Meza M., Herrera-Marschitz M. (2015). The “first hit” toward alcohol reinforcement: Role of ethanol metabolites. Alcohol Clin. Exp. Res..

[28] Berrios-Carcamo P., Quintanilla M.E., Herrera-Marschitz M., Vasiliou V., Zapata-Torres G., Rivera-Meza M. (2016). Racemic salsolinol and its enantiomers act as agonists of the mu-opioid receptor by activating the gi protein-adenylate cyclase pathway. Front. Behav. Neurosci..

[29] Berríos-Cárcamo P., Rivera-Meza M., Herrera-Marschitz M., Zapata-Torres G. (2019). Molecular modeling of salsolinol, a full gi protein agonist of the μ-opioid receptor, within the receptor binding site. Chem. Biol. Drug Design.

[30] Xie G., Hipolito L., Zuo W., Polache A., Granero L., Krnjevic K., Ye J.H. (2012). Salsolinol stimulates dopamine neurons in slices of posterior ventral tegmental area indirectly by activating mu-opioid receptors. J. Pharmacol. Exp. Ther..

[31] Szabo B., Siemes S., Wallmichrath I. (2002). Short communication inhibition of gabaergic neurotransmission in the ventral tegmental area by cannabinoids. Eur. J. Neurosci..

[32] Pons S., Fattore L., Cossu G., Tolu S., Porcu E., McIntosh J., Changeux J., Maskos U., Fratta W. (2008). Crucial role of α4 and α6 nicotinic acetylcholine receptor subunits from ventral tegmental area in systemic nicotine self-administration. J. Neurosci..

[33] Pontieri F.E., Tanda G., Orzi F., Di Chiara G. (1996). Effects of nicotine on the nucleus accumbens and similarity to those of addictive drugs. Nature.

[34] Koob G.F. (2011). Neurobiology of addiction. Focus.

[35] Lüscher C., Malenka R.C. (2011). Drug-evoked synaptic plasticity in addiction: From molecular changes to circuit remodeling. Neuron.

[36] Di Chiara G., Bassareo V. (2007). Reward system and addiction: What dopamine does and doesn’t do. Curr. Opin. Pharmacol..

[37] Kalivas P.W. (2009). The glutamate homeostasis hypothesis of addiction. Nat. Rev. Neurosci..

[38] Scofield M.D., Heinsbroek J.A., Gipson C.D., Kupchik Y.M., Spencer S., Smith A.C., Roberts-Wolfe D., Kalivas P.W. (2016). The nucleus accumbens: Mechanisms of addiction across drug classes reflect the importance of glutamate homeostasis. Pharmacol. Rev..

[39] Cornish J.L., Kalivas P.W. (2000). Glutamate transmission in the nucleus accumbens mediates relapse in cocaine addiction. J. Neurosci..

[40] Bechard A.R., Knackstedt L.A. (2019). Glutamatergic neuroplasticity in addiction. Neural Mechanisms of Addiction.

[41] Louveau A., Harris T.H., Kipnis J. (2015). Revisiting the mechanisms of cns immune privilege. Trends Immunol..

[42] Galea I., Bechmann I., Perry V.H. (2007). What is immune privilege (not)?. Trends Immunol..

[43] Colombo E., Farina C. (2016). Astrocytes: Key regulators of neuroinflammation. Trends Immunol..

[44] Kraft A.D., Harry G.J. (2011). Features of microglia and neuroinflammation relevant to environmental exposure and neurotoxicity. Int. J. Environ. Res. Public Health.

[45] Fischer R., Maier O. (2015). Interrelation of oxidative stress and inflammation in neurodegenerative disease: Role of tnf. Oxid. Med. Cell. Longev..

[46] Block M.L., Zecca L., Hong J.-S. (2007). Microglia-mediated neurotoxicity: Uncovering the molecular mechanisms. Nat. Rev. Neurosci..

[47] Herrero-Mendez A., Almeida A., Fernández E., Maestre C., Moncada S., Bolaños J.P. (2009). The bioenergetic and antioxidant status of neurons is controlled by continuous degradation of a key glycolytic enzyme by apc/c–cdh1. Nat. Cell Biol..

[48] Ren X., Zou L., Zhang X., Branco V., Wang J., Carvalho C., Holmgren A., Lu J. (2017). Redox signaling mediated by thioredoxin and glutathione systems in the central nervous system. Antioxid. Redox Signal..

[49] Van Horssen J., Van Schaik P., Witte M. (2019). Inflammation and mitochondrial dysfunction: A vicious circle in neurodegenerative disorders?. Neurosci. Lett..

[50] Cobley J.N., Fiorello M.L., Bailey D.M. (2018). 13 reasons why the brain is susceptible to oxidative stress. Redox Biol..

[51] Massaad C.A., Klann E. (2011). Reactive oxygen species in the regulation of synaptic plasticity and memory. Antioxid. Redox Signal..

[52] Hsieh H.-L., Wang H.-H., Wu W.-B., Chu P.-J., Yang C.-M. (2010). Transforming growth factor-β1 induces matrix metalloproteinase-9 and cell migration in astrocytes: Roles of ros-dependent erk-and jnk-nf-κb pathways. J. Neuroinflamm..

[53] Park J., Min J.-S., Kim B., Chae U.-B., Yun J.W., Choi M.-S., Kong I.-K., Chang K.-T., Lee D.-S. (2015). Mitochondrial ros govern the lps-induced pro-inflammatory response in microglia cells by regulating mapk and nf-κb pathways. Neurosci. Lett..

[54] Blaser H., Dostert C., Mak T.W., Brenner D. (2016). Tnf and ros crosstalk in inflammation. Trends Cell Biol..

[55] Qin L., Wu X., Block M.L., Liu Y., Breese G.R., Hong J.S., Knapp D.J., Crews F.T. (2007). Systemic lps causes chronic neuroinflammation and progressive neurodegeneration. Glia.

[56] Cahill C.M., Taylor A.M. (2017). Neuroinflammation-a co-occurring phenomenon linking chronic pain and opioid dependence. Curr. Opin. Behav. Sci..

[57] Leclercq S., De Timary P., Delzenne N.M., Stärkel P. (2017). The link between inflammation, bugs, the intestine and the brain in alcohol dependence. Transl. Psychiatr..

[58] Hofford R.S., Russo S.J., Kiraly D.D. (2019). Neuroimmune mechanisms of psychostimulant and opioid use disorders. Eur. J. Neurosci..

[59] Kohno M., Link J., Dennis L.E., McCready H., Huckans M., Hoffman W.F., Loftis J.M. (2019). Neuroinflammation in addiction: A review of neuroimaging studies and potential immunotherapies. Pharmacol. Biochem. Behav..

[60] Zhang Y., Lv X., Bai Y., Zhu X., Wu X., Chao J., Duan M., Buch S., Chen L., Yao H. (2015). Involvement of sigma-1 receptor in astrocyte activation induced by methamphetamine via up-regulation of its own expression. J. Neuroinflamm..

[61] Chao J., Zhang Y., Du L., Zhou R., Wu X., Shen K., Yao H. (2017). Molecular mechanisms underlying the involvement of the sigma-1 receptor in methamphetamine-mediated microglial polarization. Sci. Rep..

[62] Periyasamy P., Liao K., Kook Y.H., Niu F., Callen S.E., Guo M.-L., Buch S. (2018). Cocaine-mediated downregulation of mir-124 activates microglia by targeting klf4 and tlr4 signaling. Molecular Neurobiol..

[63] Pascual M., Baliño P., Aragón C.M., Guerri C. (2015). Cytokines and chemokines as biomarkers of ethanol-induced neuroinflammation and anxiety-related behavior: Role of tlr4 and tlr2. Neuropharmacology.

[64] Alfonso-Loeches S., Pascual-Lucas M., Blanco A.M., Sanchez-Vera I., Guerri C. (2010). Pivotal role of tlr4 receptors in alcohol-induced neuroinflammation and brain damage. J. Neurosci..

[65] Quintanilla M.E., Morales P., Ezquer F., Ezquer M., Herrera-Marschitz M., Israel Y. (2018). Commonality of ethanol and nicotine reinforcement and relapse in wistar-derived uchb rats: Inhibition by n-acetylcysteine. Alcohol. Clin. Exp. Res..

[66] Quintanilla M.E., Ezquer F., Morales P., Santapau D., Berríos-Cárcamo P., Ezquer M., Herrera-Marschitz M., Israel Y. (2019). Intranasal mesenchymal stem cell secretome administration markedly inhibits alcohol and nicotine self-administration and blocks relapse-intake: Mechanism and translational options. Stem Cell Res. Ther..

[67] Wang X., Loram L.C., Ramos K., De Jesus A.J., Thomas J., Cheng K., Reddy A., Somogyi A.A., Hutchinson M.R., Watkins L.R. (2012). Morphine activates neuroinflammation in a manner parallel to endotoxin. Proc. Natl. Acad. Sci. USA.

[68] Zamberletti E., Gabaglio M., Prini P., Rubino T., Parolaro D. (2015). Cortical neuroinflammation contributes to long-term cognitive dysfunctions following adolescent delta-9-tetrahydrocannabinol treatment in female rats. Eur. Neuropsychopharmacol..

[69] Cutando L., Busquets-Garcia A., Puighermanal E., Gomis-González M., Delgado-García J.M., Gruart A., Maldonado R., Ozaita A. (2013). Microglial activation underlies cerebellar deficits produced by repeated cannabis exposure. J. Clin. Investig..

[70] Munoz P., Huenchuguala S., Paris I., Segura-Aguilar J. (2012). Dopamine oxidation and autophagy. Parkinson’s Dis..

[71] Monzani E., Nicolis S., Dell’Acqua S., Capucciati A., Bacchella C., Zucca F.A., Mosharov E.V., Sulzer D., Zecca L., Casella L. (2019). Dopamine, oxidative stress and protein–quinone modifications in Parkinson’s and other neurodegenerative diseases. Angew. Chem. Int. Ed..

[72] Miyazaki I., Asanuma M. (2009). Approaches to prevent dopamine quinone-induced neurotoxicity. Neurochem. Res..

[73] D’Ambrosi N., Rossi L. (2015). Copper at synapse: Release, binding and modulation of neurotransmission. Neurochem. Int..

[74] Hastings T.G. (1995). Enzymatic oxidation of dopamine: The role of prostaglandin h synthase. J. Neurochem..

[75] Ramkissoon A., Wells P.G. (2011). Human prostaglandin h synthase (hphs)-1-and hphs-2-dependent bioactivation, oxidative macromolecular damage, and cytotoxicity of dopamine, its precursor, and its metabolites. Free Radic. Biol. Med..

[76] Meiser J., Weindl D., Hiller K. (2013). Complexity of dopamine metabolism. Cell Commun. Signal. CCS.

[77] Skrabalova J., Drastichova Z., Novotny J. (2013). Morphine as a potential oxidative stress-causing agent. Mini Rev. Org. Chem..

[78] Kuhn D.M., Francescutti-Verbeem D.M., Thomas D.M. (2006). Dopamine quinones activate microglia and induce a neurotoxic gene expression profile: Relationship to methamphetamine-induced nerve ending damage. Ann. N. Y. Acad. Sci..

[79] Sato H., Tamba M., Ishii T., Bannai S. (1999). Cloning and expression of a plasma membrane cystine/glutamate exchange transporter composed of two distinct proteins. J. Biol. Chem..

[80] Baker D.A., Xi Z.-X., Shen H., Swanson C.J., Kalivas P.W. (2002). The origin and neuronal function of in vivo nonsynaptic glutamate. J. Neurosc..

[81] Bridges R., Lutgen V., Lobner D., Baker D.A. (2012). Thinking outside the cleft to understand synaptic activity: Contribution of the cystine-glutamate antiporter (system Xc−) to normal and pathological glutamatergic signaling. Pharmacol. Rev..

[82] Baker D.A., McFarland K., Lake R.W., Shen H., Tang X.-C., Toda S., Kalivas P.W. (2003). Neuroadaptations in cystine-glutamate exchange underlie cocaine relapse. Nat. Neurosci..

[83] Madayag A., Lobner D., Kau K.S., Mantsch J.R., Abdulhameed O., Hearing M., Grier M.D., Baker D.A. (2007). Repeated n-acetylcysteine administration alters plasticity-dependent effects of cocaine. J. Neurosci..

[84] Amaral V.C.S., Morais-Silva G., Laverde C.F., Marin M.T. (2020). Susceptibility to extinction and reinstatement of ethanol-induced conditioned place preference is related to differences in astrocyte cystine-glutamate antiporter content. Neurosci. Res..

[85] Knackstedt L.A., LaRowe S., Mardikian P., Malcolm R., Upadhyaya H., Hedden S., Markou A., Kalivas P.W. (2009). The role of cystine-glutamate exchange in nicotine dependence in rats and humans. Biol. Psychiatr..

[86] Ghasemitarei M., Yusupov M., Razzokov J., Shokri B., Bogaerts A. (2019). Effect of oxidative stress on cystine transportation by xc^−^ antiporter. Arch. Biochem. Biophys..

[87] Sadakierska-Chudy A., Frankowska M., Filip M. (2014). Mitoepigenetics and drug addiction. Pharmacol. Ther..

[88] Mansouri A., Demeilliers C., Amsellem S., Pessayre D., Fromenty B. (2001). Acute ethanol administration oxidatively damages and depletes mitochondrial DNA in mouse liver, brain, heart, and skeletal muscles: Protective effects of antioxidants. J. Pharmacol. Exp. Ther..

[89] Nissanka N., Moraes C.T. (2018). Mitochondrial DNA damage and reactive oxygen species in neurodegenerative disease. FEBS Lett..

[90] Cunha-Oliveira T., Silva L., Silva A.M., Moreno A.J., Oliveira C.R., Santos M.S. (2013). Mitochondrial complex i dysfunction induced by cocaine and cocaine plus morphine in brain and liver mitochondria. Toxicol. Lett..

[91] Li N., Ragheb K., Lawler G., Sturgis J., Rajwa B., Melendez J.A., Robinson J.P. (2003). Mitochondrial complex i inhibitor rotenone induces apoptosis through enhancing mitochondrial reactive oxygen species production. J. Biol. Chem..

[92] Thangaraj A., Periyasamy P., Guo M.-L., Chivero E.T., Callen S., Buch S. (2020). Mitigation of cocaine-mediated mitochondrial damage, defective mitophagy and microglial activation by superoxide dismutase mimetics. Autophagy.

[93] Chivero E.T., Ahmad R., Thangaraj A., Periyasamy P., Kumar B., Kroeger E., Feng D., Guo M.-L., Roy S., Dhawan P. (2019). Cocaine induces inflammatory gut milieu by compromising the mucosal barrier integrity and altering the gut microbiota colonization. Sci. Rep..

[94] Narvaez J.C., Magalhães P.V., Fries G.R., Colpo G.D., Czepielewski L.S., Vianna P., Chies J.A.B., Rosa A.R., Von Diemen L., Vieta E. (2013). Peripheral toxicity in crack cocaine use disorders. Neurosci. Lett..

[95] Moreira F.P., Medeiros J.R.C., Lhullier A.C., De Mattos Souza L.D., Jansen K., Portela L.V., Lara D.R., Da Silva R.A., Wiener C.D., Oses J.P. (2016). Cocaine abuse and effects in the serum levels of cytokines il-6 and il-10. Drug Alcohol Depend..

[96] Purohit V., Bode J.C., Bode C., Brenner D.A., Choudhry M.A., Hamilton F., Kang Y.J., Keshavarzian A., Rao R., Sartor R.B. (2008). Alcohol, intestinal bacterial growth, intestinal permeability to endotoxin, and medical consequences: Summary of a symposium. Alcohol.

[97] Seitz H.K., Meier P. (2007). The role of acetaldehyde in upper digestive tract cancer in alcoholics. Transl. Res..

[98] Huang X., Li X., Ma Q., Xu Q., Duan W., Lei J., Zhang L., Wu Z. (2015). Chronic alcohol exposure exacerbates inflammation and triggers pancreatic acinar-to-ductal metaplasia through pi3k/akt/ikk. Int. J. Mol. Med..

[99] Banks W.A. (2015). The blood-brain barrier in neuroimmunology: Tales of separation and assimilation. Brain Behav. Immun..

[100] Blednov Y., Benavidez J.M., Geil C., Perra S., Morikawa H., Harris R. (2011). Activation of inflammatory signaling by lipopolysaccharide produces a prolonged increase of voluntary alcohol intake in mice. Brain Behav. Immun..

[101] Leclercq S., Cani P.D., Neyrinck A.M., Stärkel P., Jamar F., Mikolajczak M., Delzenne N.M., De Timary P. (2012). Role of intestinal permeability and inflammation in the Biol.ical and behavioral control of alcohol-dependent subjects. Brain Behav. Immun..

[102] Leclercq S., De Saeger C., Delzenne N., De Timary P., Stärkel P. (2014). Role of inflammatory pathways, blood mononuclear cells, and gut-derived bacterial products in alcohol dependence. Biol. Psychiatr..

[103] Lowe P.P., Gyongyosi B., Satishchandran A., Iracheta-Vellve A., Cho Y., Ambade A., Szabo G. (2018). Reduced gut microbiome protects from alcohol-induced neuroinflammation and alters intestinal and brain inflammasome expression. J. Neuroinflamm..

[104] Quintanilla M.E., Ezquer F., Morales P., Ezquer M., Herrera-Marschitz M., Israel Y. (2020). Innate gut microbiota is required for the acquisition of ethanol intake and relapse binge-drinking by wistar-derived high drinker rats. 43rd annual poster abstracts of the research society on alcoholism jointly with the international society for biomedical research on alcoholism, June 2020. Alcohol. Clin. Exp. Res..

[105] Northcutt A., Hutchinson M., Wang X., Baratta M., Hiranita T., Cochran T., Pomrenze M., Galer E., Kopajtic T., Li C. (2015). Dat isn’t all that: Cocaine reward and reinforcement require toll-like receptor 4 signaling. Mol. Psychiatr..

[106] Hutchinson M.R., Zhang Y., Shridhar M., Evans J.H., Buchanan M.M., Zhao T.X., Slivka P.F., Coats B.D., Rezvani N., Wieseler J. (2010). Evidence that opioids may have toll-like receptor 4 and md-2 effects. Brain Behav. Immun..

[107] Eidson L.N., Inoue K., Young L.J., Tansey M.G., Murphy A.Z. (2017). Toll-like receptor 4 mediates morphine-induced neuroinflammation and tolerance via soluble tumor necrosis factor signaling. Neuropsychopharmacology.

[108] Pan Y., Sun X., Jiang L., Hu L., Han Y., Qian C., Song C., Qian Y., Liu W. (2016). Metformin reduces morphine tolerance by inhibiting microglial-mediated neuroinflammation. J. Neuroinflamm..

[109] Yu M., Wang H., Ding A., Golenbock D.T., Latz E., Czura C.J., Fenton M.J., Tracey K.J., Yang H. (2006). Hmgb1 signals through toll-like receptor (tlr) 4 and tlr2. Shock.

[110] Zou J.Y., Crews F.T. (2014). Release of neuronal hmgb1 by ethanol through decreased hdac activity activates brain neuroimmune signaling. PLoS ONE.

[111] Crews F.T., Qin L., Sheedy D., Vetreno R.P., Zou J. (2013). High mobility group box 1/toll-like receptor danger signaling increases brain neuroimmune activation in alcohol dependence. Biol. Psychiatr..

[112] Fernandez-Lizarbe S., Montesinos J., Guerri C. (2013). Ethanol induces tlr 4/tlr 2 association, triggering an inflammatory response in microglial cells. J. Neurochem..

[113] Hutchinson M.R., Northcutt A., Hiranita T., Wang X., Lewis S., Thomas J., Van Steeg K., Kopajtic T., Loram L., Sfregola C. (2012). Opioid activation of toll-like receptor 4 contributes to drug reinforcement. J. Neurosci..

[114] Yue K., Tanda G., Katz J.L., Zanettini C. (2020). A further assessment of a role for toll-like receptor 4 in the reinforcing and reinstating effects of opioids. Behav. Pharmacol..

[115] Brown K.T., Levis S.C., O’Neill C.E., Northcutt A.L., Fabisiak T.J., Watkins L.R., Bachtell R.K. (2018). Innate immune signaling in the ventral tegmental area contributes to drug-primed reinstatement of cocaine seeking. Brain Behav. Immun..

[116] Janova H., Böttcher C., Holtman I.R., Regen T., Van Rossum D., Götz A., Ernst A.S., Fritsche C., Gertig U., Saiepour N. (2016). Cd 14 is a key organizer of microglial responses to cns infection and injury. Glia.

[117] Blednov Y.A., Black M., Chernis J., Da Costa A., Mayfield J., Harris R.A. (2017). Ethanol consumption in mice lacking cd14, tlr2, tlr4, or myd88. Alcohol. Clin. Exp. Res..

[118] Blednov Y.A., Ponomarev I., Geil C., Bergeson S., Koob G.F., Harris R.A. (2012). Neuroimmune regulation of alcohol consumption: Behavioral validation of genes obtained from genomic studies. Addict. Biol..

[119] Haydon P.G. (2001). Glia: Listening and talking to the synapse. Nat. Rev. Neurosci..

[120] Herrera-Marschitz M., You Z.B., Goiny M., Meana J., Silveira R., Godukhin O., Chen Y., Espinoza S., Pettersson E., Loidl C. (1996). On the origin of extracellular glutamate levels monitored in the basal ganglia of the rat by in vivo microdialysis. J. Neurochem..

[121] Melendez R.I., Vuthiganon J., Kalivas P.W. (2005). Regulation of extracellular glutamate in the prefrontal cortex: Focus on the cystine glutamate exchanger and group i metabotropic glutamate receptors. J. Pharmacol. Exp. Ther..

[122] Tanaka K., Watase K., Manabe T., Yamada K., Watanabe M., Takahashi K., Iwama H., Nishikawa T., Ichihara N., Kikuchi T. (1997). Epilepsy and exacerbation of brain injury in mice lacking the glutamate transporter glt-1. Science.

[123] Danbolt N.C. (2001). Glutamate uptake. Prog. Neurobiol..

[124] Robinson M. (1998). Review article the family of sodium-dependent glutamate transporters: A focus on the glt-1/eaat2 subtype. Neurochem. Int..

[125] Trotti D., Rossi D., Gjesdal O., Levy L.M., Racagni G., Danbolt N.C., Volterra A. (1996). Peroxynitrite inhibits glutamate transporter subtypes. J. Biol. Chem..

[126] Volterra A., Trotti D., Tromba C., Floridi S., Racagni G. (1994). Glutamate uptake inhibition by oxygen free radicals in rat cortical astrocytes. J. Neurosci..

[127] Trotti D., Danbolt N.C., Volterra A. (1998). Glutamate transporters are oxidant-vulnerable: A molecular link between oxidative and excitotoxic neurodegeneration?. Trends Pharmacol. Sci..

[128] Trotti D., Rizzini B.L., Rossi D., Haugeto O., Racagni G., Danbolt N.C., Volterra A. (1997). Neuronal and glial glutamate transporters possess an sh-based redox regulatory mechanism. Eur. J. Neurosci..

[129] Schaur R.J., Siems W., Bresgen N., Eckl P.M. (2015). 4-hydroxy-nonenal—A bioactive lipid peroxidation product. Biomolecules.

[130] Miralles V.J., Martínez-López I., Zaragozá R., Borrás E., García C., Pallardó F.V., Viña J.R. (2001). Na+ dependent glutamate transporters (eaat1, eaat2, and eaat3) in primary astrocyte cultures: Effect of oxidative stress. Brain Res..

[131] Lu M., Hu L.-F., Hu G., Bian J.-S. (2008). Hydrogen sulfide protects astrocytes against h2o2-induced neural injury via enhancing glutamate uptake. Free Radic. Biol. Med..

[132] Chen Y., Ying W., Simma V., Chen Y., Copin J.C., Chan P.H., Swanson R.A. (2000). Overexpression of cu, zn superoxide dismutase attenuates oxidative inhibition of astrocyte glutamate uptake. J. Neurochem..

[133] Sorg O., Horn T.F., Yu N., Gruol D.L., Bloom F.E. (1997). Inhibition of astrocyte glutamate uptake by reactive oxygen species: Role of antioxidant enzymes. Mol. Med..

[134] Muscoli C., Dagostino C., Ilari S., Lauro F., Gliozzi M., Bardhi E., Palma E., Mollace V., Salvemini D. (2013). Posttranslational nitration of tyrosine residues modulates glutamate transmission and contributes to n-methyl-d-aspartate-mediated thermal hyperalgesia. Mediat. Inflamm..

[135] Zhao W., Xie W., Le W., Beers D.R., He Y., Henkel J.S., Simpson E.P., Yen A.A., Xiao Q., Appel S.H. (2004). Activated microglia initiate motor neuron injury by a nitric oxide and glutamate-mediated mechanism. J. Neuropathol. Exp. Neurol..

[136] Zou J.Y., Crews F.T. (2005). Tnfα potentiates glutamate neurotoxicity by inhibiting glutamate uptake in organotypic brain slice cultures: Neuroprotection by nfκb inhibition. Brain Res..

[137] Szymocha R., Akaoka H., Dutuit M., Malcus C., Didier-Bazes M., Belin M.-F., Giraudon P. (2000). Human t-cell lymphotropic virus type 1-infected t lymphocytes impair catabolism and uptake of glutamate by astrocytes via tax-1 and tumor necrosis factor alpha. J. Virol..

[138] Ye Z.-C., Sontheimer H. (1996). Cytokine modulation of glial glutamate uptake: A possible involvement of nitric oxide. Neuroreport.

[139] Fine S.M., Angel R.A., Perry S.W., Epstein L.G., Rothstein J.D., Dewhurst S., Gelbard H.A. (1996). Tumor necrosis factor α inhibits glutamate uptake by primary human astrocytes implications for pathogenesis of hiv-1 dementia. J. Biol. Chem..

[140] Hu S., Sheng W.S., Ehrlich L.C., Peterson P.K., Chao C.C. (2000). Cytokine effects on glutamate uptake by human astrocytes. Neuroimmunomodulation.

[141] Chao C.C., Hu S., Ehrlich L., Peterson P.K. (1995). Interleukin-1 and tumor necrosis factor-α synergistically mediate neurotoxicity: Involvement of nitric oxide and of n-methyl-d-aspartate receptors. Brain Behav. Immun..

[142] Sitcheran R., Gupta P., Fisher P.B., Baldwin A.S. (2005). Positive and negative regulation of eaat2 by nf-κb: A role for n-myc in tnfα-controlled repression. EMBO J..

[143] Wang Z., Pekarskaya O., Bencheikh M., Chao W., Gelbard H.A., Ghorpade A., Rothstein J.D., Volsky D.J. (2003). Reduced expression of glutamate transporter eaat2 and impaired glutamate transport in human primary astrocytes exposed to hiv-1 or gp120. Virology.

[144] Moussawi K., Pacchioni A., Moran M., Olive M.F., Gass J.T., Lavin A., Kalivas P.W. (2009). N-acetylcysteine reverses cocaine-induced metaplasticity. Nat. Neurosci..

[145] LaRowe S.D., Myrick H., Hedden S., Mardikian P., Saladin M., McRae A., Brady K., Kalivas P.W., Malcolm R. (2007). Is cocaine desire reduced by n-acetylcysteine?. Am. J. Psychiatr..

[146] Moussawi K., Kalivas P.W. (2010). Group ii metabotropic glutamate receptors (mglu2/3) in drug addiction. Eur. J. Pharmacol..

[147] Moran M.M., McFarland K., Melendez R.I., Kalivas P.W., Seamans J.K. (2005). Cystine/glutamate exchange regulates metabotropic glutamate receptor presynaptic inhibition of excitatory transmission and vulnerability to cocaine seeking. J. Neurosci..

[148] Quintanilla M.E., Ezquer F., Morales P., Ezquer M., Olivares B., Santapau D., Herrera-Marschitz M., Israel Y. (2020). N-acetylcysteine and acetylsalicylic acid inhibit alcohol consumption by different mechanisms: Combined protection. Front. Behav. Neurosci..

[149] Parsegian A., See R.E. (2014). Dysregulation of dopamine and glutamate release in the prefrontal cortex and nucleus accumbens following methamphetamine self-administration and during reinstatement in rats. Neuropsychopharmacology.

[150] Lominac K.D., Sacramento A.D., Szumlinski K.K., Kippin T.E. (2012). Distinct neurochemical adaptations within the nucleus accumbens produced by a history of self-administered vs non-contingently administered intravenous methamphetamine. Neuropsychopharmacology.

[151] Griffin III W.C., Haun H.L., Hazelbaker C.L., Ramachandra V.S., Becker H.C. (2014). Increased extracellular glutamate in the nucleus accumbens promotes excessive ethanol drinking in ethanol dependent mice. Neuropsychopharmacology.

[152] Griffin W.C., Ramachandra V.S., Knackstedt L.A., Becker H.C. (2015). Repeated cycles of chronic intermittent ethanol exposure increases basal glutamate in the nucleus accumbens of mice without affecting glutamate transport. Front. Pharmacol..

[153] Ding Z.M., Rodd Z.A., Engleman E.A., Bailey J.A., Lahiri D.K., McBride W.J. (2013). Alcohol drinking and deprivation alter basal extracellular glutamate concentrations and clearance in the mesolimbic system of alcohol-preferring (p) rats. Addict. Biol..

[154] Rothstein J.D., Patel S., Regan M.R., Haenggeli C., Huang Y.H., Bergles D.E., Jin L., Hoberg M.D., Vidensky S., Chung D.S. (2005). Β-lactam antibiotics offer neuroprotection by increasing glutamate transporter expression. Nature.

[155] Lewerenz J., Albrecht P., Tien M.L.T., Henke N., Karumbayaram S., Kornblum H.I., Wiedau-Pazos M., Schubert D., Maher P., Methner A. (2009). Induction of nrf2 and xct are involved in the action of the neuroprotective antibiotic ceftriaxone in vitro. J. Neurochem..

[156] Knackstedt L.A., Melendez R.I., Kalivas P.W. (2010). Ceftriaxone restores glutamate homeostasis and prevents relapse to cocaine seeking. Biol. Psychiatr..

[157] Garcia E.J., Arndt D.L., Cain M.E. (2019). Dynamic interactions of ceftriaxone and environmental variables suppress amphetamine seeking. Brain Res..

[158] Weiland A., Garcia S., Knackstedt L.A. (2015). Ceftriaxone and cefazolin attenuate the cue-primed reinstatement of alcohol-seeking. Front. Pharmacol..

[159] Sari Y., Sakai M., Weedman J.M., Rebec G.V., Bell R.L. (2011). Ceftriaxone, a beta-lactam antibiotic, reduces ethanol consumption in alcohol-preferring rats. Alcohol Alcohol..

[160] Das S.C., Yamamoto B.K., Hristov A.M., Sari Y. (2015). Ceftriaxone attenuates ethanol drinking and restores extracellular glutamate concentration through normalization of glt-1 in nucleus accumbens of male alcohol-preferring rats. Neuropharmacology.

[161] Alshehri F.S., Hakami A.Y., Althobaiti Y.S., Sari Y. (2018). Effects of ceftriaxone on hydrocodone seeking behavior and glial glutamate transporters in p rats. Behav. Brain Res..

[162] Alajaji M., Bowers M., Knackstedt L., Damaj M. (2013). Effects of the beta-lactam antibiotic ceftriaxone on nicotine withdrawal and nicotine-induced reinstatement of preference in mice. Psychopharmacology.

[163] Aldini G., Altomare A., Baron G., Vistoli G., Carini M., Borsani L., Sergio F. (2018). N-acetylcysteine as an antioxidant and disulphide breaking agent: The reasons why. Free Radic. Res..

[164] Schneider R., Bandiera S., Souza D.G., Bellaver B., Caletti G., Quincozes-Santos A., Elisabetsky E., Gomez R. (2017). N-acetylcysteine prevents alcohol related neuroinflammation in rats. Neurochem. Res..

[165] Swanepoel T., Möller M., Harvey B.H. (2018). N-acetyl cysteine reverses bio-behavioural changes induced by prenatal inflammation, adolescent methamphetamine exposure and combined challenges. Psychopharmacology.

[166] Israel Y., Quintanilla M.E., Ezquer F., Morales P., Santapau D., Berríos-Cárcamo P., Ezquer M., Olivares B., Herrera-Marschitz M. (2019). Aspirin and n-acetylcysteine co-administration markedly inhibit chronic ethanol intake and block relapse binge drinking: Role of neuroinflammation-oxidative stress self-perpetuation. Addiction Biol..

[167] Lebourgeois S., González-Marín M.C., Jeanblanc J., Naassila M., Vilpoux C. (2018). Effect of n-acetylcysteine on motivation, seeking and relapse to ethanol self-administration. Addict. Biol..

[168] Reichel C.M., Moussawi K., Do P.H., Kalivas P.W., See R.E. (2011). Chronic n-acetylcysteine during abstinence or extinction after cocaine self-administration produces enduring reductions in drug seeking. J. Pharmacol. Exp. Ther..

[169] Spencer S., Neuhofer D., Chioma V.C., Garcia-Keller C., Schwartz D.J., Allen N., Scofield M.D., Ortiz-Ithier T., Kalivas P.W. (2018). A model of δ9-tetrahydrocannabinol self-administration and reinstatement that alters synaptic plasticity in nucleus accumbens. Biol. Psychiatr..

[170] Zhou W., Kalivas P.W. (2008). N-acetylcysteine reduces extinction responding and induces enduring reductions in cue-and heroin-induced drug-seeking. Biol. Psychiatr..

[171] Lee W.M. (2004). Acetaminophen and the us acute liver failure study group: Lowering the risks of hepatic failure. Hepatology.

[172] Duailibi M.S., Cordeiro Q., Brietzke E., Ribeiro M., LaRowe S., Berk M., Trevizol A.P. (2017). N-acetylcysteine in the treatment of craving in substance use disorders: Systematic review and meta-analysis. Am. J. Addict..

[173] Holdiness M.R. (1991). Clinical pharmacokinetics of n-acetylcysteine. Clin. Pharmacokinet..

[174] Evren C., Alniak I. (2020). N-acetylcysteine in the treatment of substance use disorders. Neurol. Sci..

[175] Tomko R.L., Jones J.L., Gilmore A.K., Brady K.T., Back S.E., Gray K.M. (2018). N-acetylcysteine: A potential treatment for substance use disorders. Curr. Psychiatr..

[176] Kawasaki A., Hoshino K., Osaki R., Mizushima Y., Yano S. (1992). Effect of ibudilast: A novel antiasthmatic agent, on airway hypersensitivity in bronchial asthma. J. Asthma.

[177] Rolan P., Hutchinson M., Johnson K. (2009). Ibudilast: A review of its pharmacology, efficacy and safety in respiratory and neurological disease. Exp. Opin. Pharmacother..

[178] Gibson L.C., Hastings S.F., McPhee I., Clayton R.A., Darroch C.E., Mackenzie A., MacKenzie F.L., Nagasawa M., Stevens P.A., MacKenzie S.J. (2006). The inhibitory profile of ibudilast against the human phosphodiesterase enzyme family. Eur. J. Pharmacol..

[179] Huang Z., Liu S., Zhang L., Salem M., Greig G.M., Chan C.C., Natsumeda Y., Noguchi K. (2006). Preferential inhibition of human phosphodiesterase 4 by ibudilast. Life Sci..

[180] Suzumura A., Ito A., Yoshikawa M., Sawada M. (1999). Ibudilast suppresses tnfα production by glial cells functioning mainly as type iii phosphodiesterase inhibitor in the cns. Brain Res..

[181] Mizuno T., Kurotani T., Komatsu Y., Kawanokuchi J., Kato H., Mitsuma N., Suzumura A. (2004). Neuroprotective role of phosphodiesterase inhibitor ibudilast on neuronal cell death induced by activated microglia. Neuropharmacology.

[182] Hutchinson M.R., Lewis S.S., Coats B.D., Skyba D.A., Crysdale N.Y., Berkelhammer D.L., Brzeski A., Northcutt A., Vietz C.M., Judd C.M. (2009). Reduction of opioid withdrawal and potentiation of acute opioid analgesia by systemic av411 (ibudilast). Brain Behav. Immun..

[183] Bell R.L., Lopez M.F., Cui C., Egli M., Johnson K.W., Franklin K.M., Becker H.C. (2015). Ibudilast reduces alcohol drinking in multiple animal models of alcohol dependence. Addict. Biol..

[184] Beardsley P.M., Shelton K.L., Hendrick E., Johnson K.W. (2010). The glial cell modulator and phosphodiesterase inhibitor, av411 (ibudilast), attenuates prime-and stress-induced methamphetamine relapse. Eur. J. Pharmacol..

[185] Rolan P., Gibbons J.A., He L., Chang E., Jones D., Gross M.I., Davidson J.B., Sanftner L.M., Johnson K.W. (2008). Ibudilast in healthy volunteers: Safety, tolerability and pharmacokinetics with single and multiple doses. Brit. J. Clin. Pharmacol..

[186] Ray L.A., Bujarski S., Shoptaw S., Roche D.J., Heinzerling K., Miotto K. (2017). Development of the neuroimmune modulator ibudilast for the treatment of alcoholism: A randomized, placebo-controlled, human laboratory trial. Neuropsychopharmacology.

[187] Worley M.J., Heinzerling K.G., Roche D.J., Shoptaw S. (2016). Ibudilast attenuates subjective effects of methamphetamine in a placebo-controlled inpatient study. Drug and alcohol dependence.

[188] Ricciotti E., FitzGerald G.A. (2011). Prostaglandins and inflammation. Arterioscler. Thromb. Vasc. Biol..

[189] Gonçalves J., Baptista S., Martins T., Milhazes N., Borges F., Ribeiro C.F., Malva J.O., Silva A.P. (2010). Methamphetamine-induced neuroinflammation and neuronal dysfunction in the mice hippocampus: Preventive effect of indomethacin. Eur. J. Neurosci..

[190] Valvassori S.S., Dal-Pont G.C., Tonin P.T., Varela R.B., Ferreira C.L., Gava F.F., Andersen M.L., Soares J.C., Quevedo J. (2019). Coadministration of lithium and celecoxib attenuates the behavioral alterations and inflammatory processes induced by amphetamine in an animal model of mania. Pharmacol. Biochem. Behav..

[191] Romano M., Cianci E., Simiele F., Recchiuti A. (2015). Lipoxins and aspirin-triggered lipoxins in resolution of inflammation. Eur. J. Pharmacol..

[192] Lindvall O., Barker R.A., Brustle O., Isacson O., Svendsen C.N. (2012). Clinical translation of stem cells in neurodegenerative disorders. Cell Stem Cell.

[193] Ding D.-C., Shyu W.-C., Lin S.-Z. (2011). Mesenchymal stem cells. Cell Transplant..

[194] Kim W.S., Park B.S., Kim H.K., Park J.S., Kim K.J., Choi J.S., Chung S.J., Kim D.D., Sung J.H. (2008). Evidence supporting antioxidant action of adipose-derived stem cells: Protection of human dermal fibroblasts from oxidative stress. J. Dermatol. Sci..

[195] Hegyi B., Kornyei Z., Ferenczi S., Fekete R., Kudlik G., Kovacs K.J., Madarasz E., Uher F. (2014). Regulation of mouse microglia activation and effector functions by bone marrow-derived mesenchymal stem cells. Stem Cells Develop..

[196] Zhang R., Liu Y., Yan K., Chen L., Chen X.-R., Li P., Chen F.-F., Jiang X.-D. (2013). Anti-inflammatory and immunomodulatory mechanisms of mesenchymal stem cell transplantation in experimental traumatic brain injury. J. Neuroinflamm..

[197] Zhong Z., Chen A., Fa Z., Ding Z., Xiao L., Wu G., Wang Q., Zhang R. (2020). Bone marrow mesenchymal stem cells upregulate pi3k/akt pathway and down-regulate nf-κb pathway by secreting glial cell-derived neurotrophic factors to regulate microglial polarization and alleviate deafferentation pain in rats. Neurobiol. Dis..

[198] Lee J.S., Hong J.M., Moon G.J., Lee P.H., Ahn Y.H., Bang O.Y., Collaborators S. (2010). A long-term follow-up study of intravenous autologous mesenchymal stem cell transplantation in patients with ischemic stroke. Stem Cells.

[199] Ryan J.M., Barry F.P., Murphy J.M., Mahon B.P. (2005). Mesenchymal stem cells avoid allogeneic rejection. J. Inflamm..

[200] Bai L., Lennon D.P., Eaton V., Maier K., Caplan A.I., Miller S.D., Miller R.H. (2009). Human bone marrow-derived mesenchymal stem cells induce th2-polarized immune response and promote endogenous repair in animal models of multiple sclerosis. Glia.

[201] Gutiérrez-Fernández M., Rodríguez-Frutos B., Ramos-Cejudo J., Otero-Ortega L., Fuentes B., Vallejo-Cremades M.T., Sanz-Cuesta B.E., Díez-Tejedor E. (2015). Comparison between xenogeneic and allogeneic adipose mesenchymal stem cells in the treatment of acute cerebral infarct: Proof of concept in rats. J. Transl. Med..

[202] Lanza C., Morando S., Voci A., Canesi L., Principato M.C., Serpero L.D., Mancardi G., Uccelli A., Vergani L. (2009). Neuroprotective mesenchymal stem cells are endowed with a potent antioxidant effect in vivo. J. Neurochem..

[203] Ohtaki H., Ylostalo J.H., Foraker J.E., Robinson A.P., Reger R.L., Shioda S., Prockop D.J. (2008). Stem/progenitor cells from bone marrow decrease neuronal death in global ischemia by modulation of inflammatory/immune responses. Proc. Natl. Acad. Sci. USA.

[204] Laroni A., De Rosbo N.K., Uccelli A. (2015). Mesenchymal stem cells for the treatment of neurological diseases: Immunoregulation beyond neuroprotection. Immunol. Lett..

[205] Israel Y., Ezquer F., Quintanilla M.E., Morales P., Ezquer M., Herrera-Marschitz M. (2017). Intracerebral stem cell administration inhibits relapse-like alcohol drinking in rats. Alcohol Alcohol..

[206] Ezquer F., Quintanilla M.E., Morales P., Ezquer M., Lespay-Rebolledo C., Herrera-Marschitz M., Israel Y. (2019). Activated mesenchymal stem cell administration inhibits chronic alcohol drinking and suppresses relapse-like drinking in high-alcohol drinker rats. Addict. Biol..

[207] Ezquer F., Morales P., Quintanilla M.E., Santapau D., Lespay-Rebolledo C., Ezquer M., Herrera-Marschitz M., Israel Y. (2018). Intravenous administration of anti-inflammatory mesenchymal stem cell spheroids reduces chronic alcohol intake and abolishes binge-drinking. Sci. Rep..

[208] Woodbury D., Schwarz E.J., Prockop D.J., Black I.B. (2000). Adult rat and human bone marrow stromal cells differentiate into neurons. J. Neurosci. Res..

[209] Meyerrose T., Olson S., Pontow S., Kalomoiris S., Jung Y., Annett G., Bauer G., Nolta J.A. (2010). Mesenchymal stem cells for the sustained in vivo delivery of bioactive factors. Adv. Drug Deliv. Rev..

[210] Caplan A.I., Correa D. (2011). The msc: An injury drugstore. Cell Stem Cell.

[211] Teixeira F.G., Carvalho M.M., Sousa N., Salgado A.J. (2013). Mesenchymal stem cells secretome: A new paradigm for central nervous system regeneration?. Cell. Mol. Life Sci. CMLS.

[212] Cantinieaux D., Quertainmont R., Blacher S., Rossi L., Wanet T., Noel A., Brook G., Schoenen J., Franzen R. (2013). Conditioned medium from bone marrow-derived mesenchymal stem cells improves recovery after spinal cord injury in rats: An original strategy to avoid cell transplantation. PLoS ONE.

[213] Saparov A., Ogay V., Nurgozhin T., Jumabay M., Chen W.C. (2016). Preconditioning of human mesenchymal stem cells to enhance their regulation of the immune response. Stem Cells Int..

[214] Stavely R., Nurgali K. (2020). The emerging antioxidant paradigm of mesenchymal stem cell therapy. Stem Cells Transl. Med..

[215] Phinney D.G., Pittenger M.F. (2017). Concise review: Msc-derived exosomes for cell-free therapy. Stem Cells.

[216] Ezquer F., Quintanilla M.E., Morales P., Santapau D., Ezquer M., Kogan M.J., Salas-Huenuleo E., Herrera-Marschitz M., Israel Y. (2019). Intranasal delivery of mesenchymal stem cell-derived exosomes reduces oxidative stress and markedly inhibits ethanol consumption and post-deprivation relapse drinking. Addict. Biol..

[217] Bushati N., Cohen S.M. (2007). Microrna functions. Annu. Rev. Cell Dev. Biol..

[218] Ha M., Kim V.N. (2014). Regulation of microrna biogenesis. Nat. Rev. Mol. Cell Biol..

[219] Guo H., Ingolia N.T., Weissman J.S., Bartel D.P. (2010). Mammalian micrornas predominantly act to decrease target mrna levels. Nature.

[220] Huntzinger E., Izaurralde E. (2011). Gene silencing by micrornas: Contributions of translational repression and mrna decay. Nat. Rev. Genet..

[221] Lim L.P., Lau N.C., Garrett-Engele P., Grimson A., Schelter J.M., Castle J., Bartel D.P., Linsley P.S., Johnson J.M. (2005). Microarray analysis shows that some micrornas downregulate large numbers of target mrnas. Nature.

[222] Bader A.G., Brown D., Winkler M. (2010). The promise of microrna replacement therapy. Cancer Res..

[223] Pottoo F.H., Javed N., Rahman J., Abu-Izneid T., Khan F.A. (2020). Targeted delivery of mirna based therapeuticals in the clinical management of glioblastoma multiforme. Seminars in Cancer Biology.

[224] Magenta A., Greco S., Gaetano C., Martelli F. (2013). Oxidative stress and micrornas in vascular diseases. Int. J. Mol. Sci..

[225] Liu G., Abraham E. (2013). Micrornas in immune response and macrophage polarization. Arterioscler. Thromb. Vasc. Biol..

[226] Saba R., Gushue S., Huzarewich R.L., Manguiat K., Medina S., Robertson C., Booth S.A. (2012). Microrna 146a (mir-146a) is over-expressed during prion disease and modulates the innate immune response and the microglial activation state. PLoS ONE.

[227] Kong H., Yin F., He F., Omran A., Li L., Wu T., Wang Y., Peng J. (2015). The effect of mir-132, mir-146a, and mir-155 on mrp8/tlr4-induced astrocyte-related inflammation. J. Mol. Neurosci..

[228] Freilich R.W., Woodbury M.E., Ikezu T. (2013). Integrated expression profiles of mrna and mirna in polarized primary murine microglia. PLoS ONE.

[229] Butovsky O., Jedrychowski M.P., Cialic R., Krasemann S., Murugaiyan G., Fanek Z., Greco D.J., Wu P.M., Doykan C.E., Kiner O. (2015). Targeting mi r-155 restores abnormal microglia and attenuates disease in sod 1 mice. Ann. Neurol..

[230] Cardoso A.L., Guedes J.R., Pereira de Almeida L., Pedroso de Lima M.C. (2012). Mir-155 modulates microglia-mediated immune response by down-regulating socs-1 and promoting cytokine and nitric oxide production. Immunology.

[231] Tarassishin L., Loudig O., Bauman A., Shafit-Zagardo B., Suh H.S., Lee S.C. (2011). Interferon regulatory factor 3 inhibits astrocyte inflammatory gene expression through suppression of the proinflammatory mir-155 and mir-155. Glia.

[232] Korotkov A., Broekaart D.W., Van Scheppingen J., Anink J.J., Baayen J.C., Idema S., Gorter J.A., Aronica E., Van Vliet E.A. (2018). Increased expression of matrix metalloproteinase 3 can be attenuated by inhibition of microrna-155 in cultured human astrocytes. J. Neuroinflamm..

[233] Cardoso A.L., Guedes J.R., De Lima M.C.P. (2016). Role of micrornas in the regulation of innate immune cells under neuroinflammatory conditions. Curr. Opin. Pharmacol..

[234] Lagos-Quintana M., Rauhut R., Yalcin A., Meyer J., Lendeckel W., Tuschl T. (2002). Identification of tissue-specific micrornas from mouse. Curr. Biol..

[235] Ponomarev E.D., Veremeyko T., Barteneva N., Krichevsky A.M., Weiner H.L. (2011). Microrna-124 promotes microglia quiescence and suppresses eae by deactivating macrophages via the c/ebp-α–pu. 1 pathway. Nat. Med..

[236] Friedman A. (2007). Transcriptional control of granulocyte and monocyte development. Oncogene.

[237] Konovalova J., Gerasymchuk D., Parkkinen I., Chmielarz P., Domanskyi A. (2019). Interplay between micrornas and oxidative stress in neurodegenerative diseases. Int. J. Mol. Sci..

[238] Juźwik C.A., Drake S.S., Zhang Y., Paradis-Isler N., Sylvester A., Amar-Zifkin A., Douglas C., Morquette B., Moore C.S., Fournier A.E. (2019). Microrna dysregulation in neurodegenerative diseases: A systematic review. Prog. Neurobiol..

[239] Henry R.J., Doran S.J., Barrett J.P., Meadows V.E., Sabirzhanov B., Stoica B.A., Loane D.J., Faden A.I. (2019). Inhibition of mir-155 limits neuroinflammation and improves functional recovery after experimental traumatic brain injury in mice. Neurotherapeutics.

[240] Rossetto I., Cagnon V., Lizarte F., Tirapelli L., Tirapelli D., Arantes R., Chuffa L., Martinez F., Martinez M. (2019). Ethanol and caffeine consumption modulates the expression of mirnas in the cerebellum and plasma of uchb rats. Life Sci..

[241] Lippai D., Bala S., Csak T., Kurt-Jones E.A., Szabo G. (2013). Chronic alcohol-induced microrna-155 contributes to neuroinflammation in a tlr4-dependent manner in mice. PLoS ONE.

[242] Bala S., Marcos M., Kodys K., Csak T., Catalano D., Mandrekar P., Szabo G. (2011). Up-regulation of microrna-155 in macrophages contributes to increased tumor necrosis factor α (tnfα) production via increased mrna half-life in alcoholic liver disease. J. Biol. Chem..

[243] Lewohl J.M., Nunez Y.O., Dodd P.R., Tiwari G.R., Harris R.A., Mayfield R.D. (2011). Up-regulation of micrornas in brain of human alcoholics. Alcohol. Clin. Exp. Res..

[244] Novo-Veleiro I., González-Sarmiento R., Cieza-Borrella C., Pastor I., Laso F.-J., Marcos M. (2014). A genetic variant in the microrna-146a gene is associated with susceptibility to alcohol use disorders. Eur. Psychiatr..

[245] Guo M.-L., Periyasamy P., Liao K., Kook Y.H., Niu F., Callen S.E., Buch S. (2016). Cocaine-mediated downregulation of microglial mir-124 expression involves promoter DNA methylation. Epigenetics.

[246] Chandrasekar V., Dreyer J.-L. (2009). Micrornas mir-124, let-7d and mir-181a regulate cocaine-induced plasticity. Mol. Cell. Neurosci..

[247] Bahi A., Dreyer J.L. (2013). Striatal modulation of bdnf expression using micro rna 124a-expressing lentiviral vectors impairs ethanol-induced conditioned-place preference and voluntary alcohol consumption. Eur. J. Neurosci..

[248] Chandrasekar V., Dreyer J.-L. (2011). Regulation of mir-124, let-7d, and mir-181a in the accumbens affects the expression, extinction, and reinstatement of cocaine-induced conditioned place preference. Neuropsychopharmacology.

